# Living standards and the life cycle: reconstructing household income and consumption in the early twentieth‐century Netherlands^†^

**DOI:** 10.1111/ehr.12997

**Published:** 2020-06-16

**Authors:** Corinne Boter

**Affiliations:** ^1^ Utrecht University

## Abstract

Conventional methods of measuring historical household living standards are often criticized because of the omission of women's and children's wages and non‐wage income; the focus on urban centres; and the exclusion of life‐cycle changes in household composition, income, and consumption. This article presents a method that accounts for these issues and applies it to agricultural and textile households in the early‐twentieth century Netherlands. It uses total household income, as opposed to the husband's wage, as the enumerator for calculating alternative welfare ratios. The results show that welfare ratios were not only structurally higher than those based on the male‐breadwinner model, but also followed a different life‐cycle trajectory. Furthermore, household portfolios were diversified and depended on local labour market structures. Thus, the study concludes that analyses based on men's wages only reflect the rough outlines of how households functioned.

During the past two decades, methods to measure historical living standards have improved significantly. In particular, Allen's welfare ratios, which show whether one man's urban wage could support himself, his wife, and two children, are widely used.^1^ However, the accuracy of this method is being questioned because of its omission of women's and children's wages and non‐wage income, its exclusive focus on urban centres, and its exclusion of life‐cycle changes in household composition, income, and consumption. An increasing body of literature is attempting to adjust the welfare ratio method to solve these issues.^2^ Following these studies, the current article presents a method that uses household income instead of men's wages to calculate welfare ratios, and takes life‐cycle changes into account. It applies this methodology to Dutch agricultural and textile households in the early twentieth century.

The Netherlands make an interesting case study for two reasons. First, virtually all agricultural wage labourers complemented their wage income by working their own land. Likewise, in the region of Twente, many people had begun to work in one of the region's many textile factories by the end of the nineteenth century, but agriculture still constituted a significant part of their household income. The survival of proto‐industrial traditions in this region is illustrative of the tight link between developments in agriculture and industry. Second, the Netherlands have long been portrayed as the ‘first male breadwinner society’.^3^ However, recent studies have demonstrated that women and children were in fact very active in the labour market by 1900,^4^ but their relative contributions to household income have remained understudied.

This article uses qualitative and quantitative information from surveys conducted around 1900 to determine the absolute and relative importance of land and wage labour by men, women, and children for agricultural and textile households. Incomes from land are quantified by determining the size of the land available and the extent and consumer price of the yields. To determine the importance of wage labour, gender‐ and age‐specific labour force participation and wage rates in agriculture and the textile industry are assessed. On the basis of this empirical foundation, welfare ratios are composed for a fixed family size, as well as for the various stages of the household life cycle.

The results lead to the following conclusions. First, for most working‐class families, land was a stable and indispensable source of income, especially for agricultural wage labourers. Incomes from land were an important buffer against misfortune, such as unexpected unemployment, and are thus a crucial factor in understanding households’ livelihood strategies. Second, women's and children's wage labour was especially extensive in textile regions where their labour was in high demand. In rural regions, their wage labour was common too, but seasonal, because labour demand was low during a significant part of the year. Thus, local labour market structures were an important factor in determining women's and children's labour force participation and wages. Third, men's wages in both agriculture and the textile industry were not high enough to sustain their family at a respectable level of existence. This corroborates prior studies that have argued that the male breadwinner model was not (yet) the dominant type of household labour division for the early twentieth‐century Dutch working class.

The rest of this article is organized as follows. Section I discusses the welfare ratio method and its pitfalls. Section II presents a brief overview of the structure of the early twentieth‐century Dutch economy and introduces the sources and methodology. Sections III and IV quantify the absolute value of land and wage labour in agriculture and the textile industry. Section V uses these findings to reconstruct the income composition and welfare ratios for two case‐study households, one agricultural and one textile, of a fixed family size, while section VI considers how they changed over the course of the family life cycle. The final section offers some conclusions.

## I

Allen's welfare ratio method is widely used by scholars investigating the long‐term development of living standards. A welfare ratio is calculated by dividing one man's unskilled urban wage by the costs of living of a man, a woman, and two children. Men's wages are used as the numerator because they are available for many regions of the world during the past 500 years, which facilitates regional and temporal comparisons. Furthermore, economic theory suggests that unskilled urban wages represent the marginal product of labour in an economy and are therefore a good indicator of general trends in living standards.^5^ Another advantage of the welfare ratio method is that it uses more realistic consumption baskets to deflate income, unlike earlier studies that used grain prices.^6^

Notwithstanding the evident benefits of the welfare ratio method, it leaves many unanswered questions about how households functioned. For example, how did families with welfare ratios below 1 survive? What about households living outside large urban centres? Indeed, criticism of this method is being articulated in a growing number of studies.^7^ They point out that, first, *household income* was diversified and the husband's (wage) labour was rarely the only source of income. Many factories of the industrial revolution were filled with women and children, who were wage earners just like their husbands and fathers. Furthermore, non‐wage income, such as the cultivation of land or running a family business, was often an important resource. Moreover, the composition and relative importance of these incomes were not constant over time and depended on the level of men's wages, local labour market structures, employment opportunities, and social norms, among other things.^8^ Thus, households’ access to resources was temporally and regionally dependent.

Second, conventional methods of calculating living standards simplify *household consumption*. The translation from individual into household consumption needs is based on flawed assumptions about family size. The average working‐class family included more children than the two ascribed to Allen's standardized household. Furthermore, unequal intra‐household resource allocation was common, which means that not every household member enjoyed the same standard of living. Men and boys were commonly privileged, consuming more and better foodstuffs than their female family members.^9^ Moreover, men did not always pool their earnings into the household budget but instead spent part of their wages in taverns. For instance, a study on family consumption in Spain between 1850 and 1930 has shown that excessive alcohol consumption ‘was not only a health problem, but also a serious economic problem for working families, affecting especially the wife—“circled by unhappy children … exhausted by insufficient nutrition”—because her husband had spent the weekly wage’.^10^

Third, neither income nor consumption were constant during the *household life cycle*. Welfare ratios decreased from the moment children started to arrive in the household. After all, more mouths needed to be fed and women's wage‐earning opportunities were likely to decline. When the children reached working age they could contribute to the household income, after which living standards increased again. Therefore, conclusions based on a standardized family size can be misleading, because the same household could have endured periods of higher and lower living standards during the various stages of its existence.^11^

The body of literature that moves beyond the use of men's urban wages to study living standards has been growing steadily over the past years. De Haas has argued for colonial and early post‐colonial Uganda that only a small share of the population worked for wages in an urban centre, and has developed an alternative methodology to capture *rural* living standards. He identified three typical Ugandan smallholdings and for each of these determined the number of consumer units, the total acreage under cultivation (divided into land for staple crops, protein crops, and cash crops), and available livestock. Subsequently, the welfare ratios of these smallholdings were calculated, with the total income from land and livestock as the numerator and the costs of the required number of rural bare‐bones subsistence baskets as the denominator. This methodological refinement reveals that the welfare ratios of the three smallholdings and those of unskilled wage labourers in Kampala (Uganda's capital) developed differently during the period 1915–70.^12^

Even though the industrialized economies of nineteenth‐century western Europe relied on wage labour significantly more than the rural economies of colonial and early post‐colonial Africa, similar objections against using men's urban wages for research on household living standards apply. As early as 1995, Horrell and Humphries showed on the basis of 1,781 British household budgets from 1787 to 1865 that the income composition of households in which the husband worked in high‐wage agriculture, low‐wage agriculture, mining, a factory, outwork, and trades developed differently over time. They concluded that ‘the variation of women's and children's contribution over time and across occupations is not consistently related to family income level’ and that local demand for labour determined women's and children's access to (wage) work.^13^

Schneider has reconstructed the welfare ratios of households in pre‐modern England for various stages of the life cycle. He determined the total caloric requirements of each family member for every stage of the life cycle, including the extra costs of pregnancy and lactation. He then constructed four families, each with a different number of births and deaths, and different birth intervals, but all with a southern English building labourer as the sole breadwinner. In only one of these four hypothetical families, he allowed the wife to earn an income of 50 per cent of her husband's wage after he died. As could be expected, welfare ratios decreased during the early stages of the life cycle when children were born into the household and started to increase again after children started to leave the household. However, Schneider's use of the male breadwinner model is problematic, as he himself acknowledged, because of ‘women's important household, non‐market, and waged work’.^14^

As mentioned in the introduction, the early twentieth‐century Netherlands makes an interesting case for further research on life‐cycle changes in the living standards of non‐urban households. This is, first, because of its economic structure, with a relatively large primary sector and the survival of proto‐industrial traditions in industrializing regions, and second, due to the much‐contested notion of the Netherlands as ‘the first male breadwinner society’.

## II

During the first half of the nineteenth century, wage labour, as opposed to independent farming, gained importance in Dutch agriculture. However, from the 1880s onwards, a process of de‐proletarianization started due to the agricultural crisis (1878–1895) which motivated farmers to invest in labour‐saving production methods. Furthermore, the demand for products that were typically cultivated on small‐scale farms (such as vegetables and fruit) increased. Because the demand for wage labour decreased as a result, many agricultural wage workers started their own business—often combined with wage labour—or migrated to the cities to find employment in the growing industrial sector.^15^

Dutch industrialization took off late relative to neighbouring countries such as Belgium and the UK, and the agricultural sector remained large well into the twentieth century. Industrialization started in the 1860s in the textile industry, of which the eastern region of Twente was the beating heart. This region had a long history of linen textile production during the early modern period, albeit mostly for domestic use. Due to the increasing influx of cotton in the European market after 1750, the linen industry suffered a major crisis from which it did not recover and most textile workers changed to working with cotton instead.^16^

The year 1830 was a pivotal moment for the Twente cotton industry when the southern provinces seceded from the Netherlands to become the independent kingdom of Belgium. The import of Flemish cotton cloth was restricted soon after, which was highly problematic since the Dutch Trading Company (Nederlandsche Handel‐Maatschappij (NHM), a state‐supported institution) exported large amounts of cotton yarn and cloth to the Dutch East Indies—the most important Dutch colony, present‐day Indonesia.^17^ To remedy this loss, the state and the NHM started to stimulate cotton textile manufacturing in Twente. To this end, weaving schools were founded to teach both men and women how to use the flying shuttle—a device that doubled a handloom weaver's output. During the first half of the nineteenth century, weaving and spinning remained largely home‐based, but this changed after 1865 when steam‐driven machinery started to dominate the production process.^18^

Developments in agriculture and (especially the textile) industry were closely intertwined. The textile workers in Twente had originally been farmers or agricultural wage labourers who spun and wove during the winter months to generate extra income during this slack period of agricultural work. Moreover, this by‐employment became ever more vital for the livelihoods of the Twente population during the seventeenth and eighteenth centuries because population growth caused increasing pressure on arable land. Proto‐industrialization, as such domestic mass production in combination with agricultural activities is often called, thus was an essential component of the functioning of the Twente economy and would, as will be demonstrated quantitatively in the next section, remain so until the start of the twentieth century.^19^

Another important reason to use the early twentieth‐century Netherlands as a case study is the much‐debated topic of the extent of female labour force participation. For a long time, historians have argued that the Netherlands was ‘the first male breadwinner society’, with low female labour force participation rates relative to other north‐western European countries even during the early modern period.^20^ They explained this trend as a result of strong social norms about domesticity, which required women to devote their time to homemaking and child rearing. However, recent research has shown that in reality, Dutch women were very active in the labour market until at least the first decades of the twentieth century.^21^ These conflicting findings can be explained by the fact that former studies were largely based on occupational censuses, which under‐registered women's labour due to their widespread involvement in part‐time, ‘assisting’, and informal labour.^22^ However, while valuable attempts have been undertaken to reconstruct female labour force participation and wage rates,^23^ the relative importance of their work for the household income has remained understudied. The same goes for children's (wage) labour and subsistence agriculture.

The current article studies the living standards of agricultural and textile households based on a *household‐income* model as opposed to a male‐breadwinner model. It presents empirical evidence on land use, and gender‐ and age‐specific labour force participation and wage rates from a wide range of sources. Most importantly, but not exclusively, three surveys from the late nineteenth and early twentieth century have been consulted. First, the 1890 labour surveys contain hundreds of interviews with labourers, entrepreneurs, vicars, mayors, and so on, to investigate whether the 1889 Labour Law fulfilled its aims.^24^ These individual accounts provide a rare insight into the daily lives of working‐class households in Twente. Second, two reports on the state of the home industry have been consulted: a catalogue of an exhibition of Dutch home industry (1909) and a nation‐wide survey (1914) for which thousands of home‐working families were visited.^25^ Both surveys provide quantitative and qualitative information about the number, age, and wages of the home workers.^26^ Third, in 1908, a detailed survey on the living standards of agricultural labourers was published, which contains information about the wages and land use of agricultural households, and the division of labour within them.^27^

## III

A vast body of qualitative evidence confirms that land was an invaluable resource for a large share of the Dutch population at the start of the twentieth century. Agricultural wage labourers often rented a small plot of land from their employer, which they used for the cultivation of potatoes and vegetables, and for keeping animals.^28^ Some were assisted by agricultural cooperatives, which facilitated the use of machinery, the acquisition of (artificial) fertilizers, forage, and sowing‐seeds, and provided credit and insurance.^29^ Others could count on the help of their employer regarding the required equipment.^30^ Married women and children played an important role in this self‐employed agriculture. The State Commission of Agriculture concluded in 1912 for the central region of De Betuwe that ‘while wage labour by women and children has decreased … the labour of these people in their own, small businesses has increased, which relates to the strong expansion of potato‐ and sugar beet cultivation by small farmers in their expanding businesses’.^31^

For textile households too, land was an important addition to the household income. A weaver from Enschede stated in 1890 that ‘[my wife] takes care of the household, in addition we cultivate a significant plot of land just outside the city … Without that land, we would not be able to get by with just weaving’.^32^ The combination of textile labour with working the land was a tradition inherited from proto‐industrial times and has been thoroughly researched.^33^ Hendrickx has illustrated the close relationship between agriculture and industry with the example of a strike in Borne in 1905 when weavers protested against the plan to extend the working day: ‘They [the weavers] argued that the longer hours would no longer enable them to work their fields in the evening, because they would be too tired’.^34^ By 1920, most textile labourers were still tied to agricultural practices and even reorientated most of their labour time to the agricultural sector later in life.^35^

Even though there is a broad consensus that land was an important part of households’ portfolios, its absolute and relative importance for the household income have never been quantified systematically. To achieve this, three steps are required. First, we need to assess the rent and the size of the land and the number of livestock available to both case‐study households.^36^ A typical agricultural wage labourer rented 73 *ares* (0.73 hectares) of land from his employer.^37^ The national average rent of one hectare was 46.80 Dutch guilders (fl.) in the period 1906–10, meaning that an agricultural household paid 34.16 fl. per year.^38^ There were regional differences in how this land was used, but the cultivation of potatoes and the keeping of goats and chickens were common throughout the entire country.^39^ Goats—‘the dairy cow of the poor’^40^—were particularly popular because they provided milk and did not demand forage or pasture as they were happy to consume leftovers from the kitchen. Bieleman has estimated that in the province of Drenthe, agricultural wage labourers’ households on average owned 1.7 goats and 12 chickens.^41^

The land use of textile labourers is not as systematically documented. A respondent in the 1890 labour survey stated that ‘[h]ouses in Enschede, at least in the outer quarters, usually come with a plot of land of 1 “spint”—200 à 250 m^2^—and not seldom with 2 “spints” or more’.^42^ The plots of land that labourers rented just outside of town were either 5 or 10 *are*.^43^ The price of land was high relative to the countryside. For Almelo it was reported that ‘200 hectares, rented to labourers in small plots, are worth 20,000 fl. in rent, this is about as much as 1,800 hectares that are rented by farmers in the countryside’.^44^ This means that one *are* of land cost 1.00 fl. per year. In sum, textile households cultivated 12.5 *are* of land (of which five came with the house and 7.5 were rented), for which they paid 12.50 fl. per year in 1890. Furthermore, these households also commonly kept goats and chickens.^45^

The second step is to calculate the average yields from land and livestock and their consumer prices. It is assumed that all the land was used for potato cultivation, goats for milk, and chickens for eggs. The national average potato production per hectare during the period 1901–10 was 201 hectolitres.^46^ Chickens on average produced 177 eggs annually and one goat could provide around three litres of milk per day (1,095 litres per year). The kind and extent of the consumer products that land and livestock yielded are summarized in table 1.

**Table 1:** Annual profits from potato cultivation and livestock in guilders, *c*. 1900

Source of income	Consumer product	Output per year	Average consumer price, 1906–10
1 hectare of land	Potatoes (consumption)	201 hectolitres	487.63
1 goat	Milk	1,095 litres	78.53^a^
1 chicken	Eggs	177 pieces	7.29^b^

*Notes: a* Price of sweet cow's milk. NB: sweet milk was more expensive than buttermilk. I use the market price of sweet milk because buttermilk was a byproduct of sweet milk and of inferior quality. The milk that households got from their own cow or goat was therefore the higher quality sweet milk.

*b* Market price.

*Sources*: Bieleman, *Boeren op het Drentse zand*, p. 516; Anonymous, ‘Hoe doet u het?’, *De Geitehouder*, 20 (1957), p. 86. Prices are taken from van Riel, *Trials of convergence*, pp. 738–49.

The final step is to account for transaction costs. The use of consumer prices is justified if households consumed everything themselves because that is the price they would have otherwise paid.^47^ However, agricultural households regularly *sold* part of their yields to local factories, while textile labourers rarely did so.^48^ To correct for transaction costs it is assumed that agricultural households cultivated as many potatoes for their own consumption as the textile households and that they used the rest of their land for the cultivation of ‘factory potatoes’ that were sold to and processed in factories for the production of potato starch.^49^ During the period 1906–10, farmers received 0.92 fl. per hectolitre of factory potatoes. All the collected information about the value of land is summarized in table 2.

**Table 2:** Value of the annual yields from self‐employed agriculture, *c*. 1910

	Agricultural households				Textile households			
Source of income	Extent	Costs (f)	Yields (f)	Profits (f)	Extent	Costs (f)	Yields (f)	Profits (f)
Land (hectares)	0.73	34.16	234.87^a^	200.71	0.125	14.15	60.95	46.80
Goat	1.7	10.76	133.51	122.75	1	6.33	78.53	72.20
Chicken	12	9.00	87.47	78.47	1	0.75	7.29	6.54
Total	‐	53.92	455.84	401.92	‐	21.23	146.78	125.55

*Notes*: Prices have been converted into 1910 guilders using the tool available at International Institute of Social History, ‘Value of the guilder/euro’, http://www.iisg.nl/hpw/calculate.php (Jan. 2019). Original prices and years are as follows: one goat = 6.50 fl. (1881); one chicken = 0.77 fl. (1881); 1 *are* of land for urban households = 1 fl. per year (1890). For the consumer prices of potatoes, milk, and eggs, see tab. 1. Due to rounding, totals might not reflect the exact sum of the presented numbers.

*a* The value of potato cultivation entails 25 hectolitres of potatoes for consumption (consumer price) and 189.37 hectolitres of factory potatoes to be sold to the potato‐starch factories (wholesale price). On one hectare of land, more factory potatoes could be cultivated than consumption potatoes (in hectolitres). I have accounted for this with help of the agricultural reports: Departement van Landbouw Nijverheid en Handel, *Verslag*, p. 51.

*Sources*: See footnotes to section III.

## IV

Having established the average incomes from self‐employed agriculture, we now have to determine gender‐ and age‐specific labour force participation and wage rates in agriculture and the textile industry. For agriculture, determining the number of working days is a challenge as seasonal unemployment was common. For men, it mattered a great deal whether they worked on fixed or casual contracts. In the former case, money wages were lower, but work was more secure and in‐kind payments—such as the use of land—were more extensive. Casual labourers’ wages were higher, mainly because they usually worked for piece rates, but they were in greater danger of becoming unemployed during the winter or when weather conditions were unfavourable.^50^

Seasonal unemployment was a far larger problem for women who were usually only hired from April to September. From April to June, they were weeding, in July labour demand briefly decreased, and during the harvest period—August and September—they worked alongside their husbands: ‘a man reaped the grain with a scythe, while a woman tied the straw together into sheaves and ordered these into bundles’.^51^ Van Nederveen Meerkerk and Paping have estimated for the province of Groningen that women worked 100 days per year for wages in 1909 (as opposed to 115 days in 1840 and 140 in 1860).^52^ Thus, women's wage labour was limited, but they spent a lot of time on their own land. The 1906 agricultural survey concluded that ‘[many of the people we questioned] called to our attention the heavy tasks of married women … who, besides fulfilling their domestic duties, are expected to carry a large share of the burden of keeping the family business running’.^53^

The absolute number of children working in agriculture was significant around 1900 as well, although the annual number of working days decreased during the second half of the nineteenth century and was concentrated in the summer months.^54^ Besides the falling demand for wage labourers, increasing school attendance also played a role in the reduction of children's working days.^55^ However, attendance rates depended on agricultural cycles as children were expected to assist their parents during the peak season. In fact, most families could not do without their children's earnings and the Law on Compulsory Education (implemented in 1901) was frequently violated.^56^ As the 1909 report concluded, ‘[w]e have not found one example of complete absence of labour among the children who are by law obliged to be in school’.^57^ Thus, school did not pull children out of the labour force entirely but it did reduce their annual working time. Seeing that older children were hired for the same kind of work as women, for them too a working year of 100 days of wage labour is chosen; that is, additional to working on the family farm. Younger children, however, seem to have been *assisting* their parents in their wage labour or on the family farm rather than working for wages independently.

**Figure 1:**
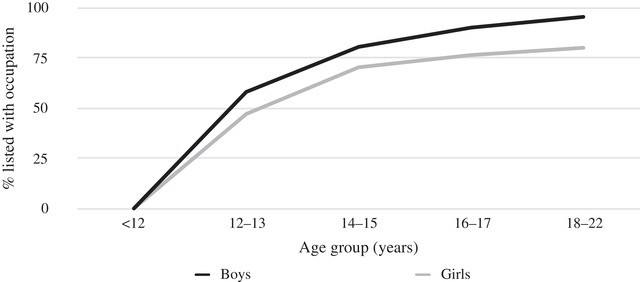
Children's age‐specific labour force participation in Enschede, 1899 *Source*: Centraal Bureau voor de Statistiek, ‘Uitkomsten der beroepstelling’.

Enschede was one of the most important textile centres in the Netherlands, its factories employing a significant share of the city's population.^58^ The labour force participation of adult men was stable throughout all age groups. Conversely, the composition of the female and child labour force showed clear life‐cycle effects. Children younger than 12 were legally not allowed to work in factories since the Child Labour Act (Kinderwetje van Van Houten) was implemented in 1874.^59^ Older children—at least in Enschede—combined wage work with attending the so‐called ‘factory school’ that provided education to employees between 12 and 16 years old. To be admitted, children were required to have a primary school diploma, which was a strong incentive for parents to send their children to school until they were 12. Figure 1 clearly illustrates this life‐cycle effect: (formal) labour force participation of boys and girls in Enschede was virtually non‐existent for the age group younger than 12 years, but shot up to 58 per cent for boys and 47 per cent for girls in the 12–13 age group and continued to increase thereafter.

Married women were not entirely absent from the factory floor, but they were the exception. The 1899 census listed 27 per cent of the married women in Enschede in the 18–22 age group with an occupation, but less than 10 per cent for older age groups. For young mothers, factory labour was particularly hard to combine with domestic duties, although there were several ways to outsource care for their children: older children could take care of their siblings and some factories provided day care. In the 1890 labour surveys several respondents mentioned that such nurseries existed in the textile factories of Almelo. However, to the question of whether everyone could take their children to these crèches, a weaver responded: ‘[the nurseries] are only for the children of widows. It occasionally occurs that a man, whose wife has died, brings his child here, but that is an exception’.^60^

Two other sources corroborate that married women were virtually absent from the factories. First, for the factory census of 1889, 3,053 factories were inspected to count the number of factory labourers divided by their sex, marital status, and age.^61^ In total, 124,934 labourers were enumerated, of which 69 per cent were male, 8 per cent female (2 per cent married and 6 per cent unmarried), and 23 per cent children (boys and girls aged 12 to 18 years).^62^ Second, a nation‐wide investigation from 1911 concluded that out of all the 304,437 Dutch factory labourers, 43,844 were women older than 16 years, of whom 4,227 (1.4 per cent of the total labour force) were married.^63^

Home‐industrial work provided married women with the opportunity to work for wages at home. A factory director from Rijssen (Twente) mentioned that he employed about 200 households to sew sacks at home and stated that women could earn a decent amount of money without having to neglect their homemaking duties.^64^ A producer from Almelo (Twente) said that there was an abundant supply of homeworkers, stating that ‘[married women] gladly want to perform some work at home to earn a little extra … the larger the household, the less time they have for extra work … Seeing that these people put so much effort into earning extra money, it is apparent that wages are not high’.^65^ By the 1910s, home industry was still important, so much so that the government conducted a nation‐wide survey on the state of home industry. The survey included 11,381 men, 8,442 women, and 3,565 children younger than 16 (1,592 boys and 1,973 girls).^66^ There was a sharp rise in the number of home‐industrial labourers after the age of 24, which implies that the major share of the home workers were married.^67^

Having established the type of work men, women, and children typically did, we now need to explore gender‐ and age‐specific wage rates. Agricultural wages depended on the age and the sex of the worker but also on the type of work, the season, and the region. In 1913, the Dutch Royal Agriculture Society (Koninklijke Nederlandsche Landbouwvereeniging) reported that male wage labourers on average earned 379.27 fl. per year, with the lowest wage (325.00 fl.) paid in Drenthe and the highest (537.70 fl.) in North Holland. These wages included in‐kind payments (such as the use of land or pasture), excluded the earnings of other household members, and accounted for seasonal unemployment.^68^ It remains unclear how many days of labour were needed to earn these wages.

For women and children, most sources only recorded daily wages that depended on the season and the type of work. Moreover, information is scattered and nation‐wide or provincial averages were seldom reported. As mentioned earlier, women and children were weeding (April to June) and assisting with the harvest (August and September). Several sources reported a woman's day wage of 0.60 fl. for weeding and 1.00 fl. per day for ‘harvest work’.^69^ The reported child wages ranged from 0.15 fl. to 0.53 fl. per day, with an average of 0.37 fl. A respondent from Holwerd (Friesland) stated that ‘[c]hildren work along, both for their parents and for wages. They perform the same work as women, their wage is 30–40 cents per day’.^70^ Based on this information, this study estimates that female wage labourers earned approximately 76.00 fl. per year (60 days of weeding and 40 days of harvest work) and children 37.00 fl.

To determine wage rates in the textile industry, we have to distinguish between factory labour and home‐industrial labour. In 1908, the Dutch Bureau of Statistics (Centraal Bureau voor de Statistiek; CBS) collected the wages of 11,861 men and 6,678 women working in the cotton factories of Twente, earning an average day wage of 1.51 fl. and 1.05 fl. respectively.^71^ Labourers in the spinning mills as well as in the weaving factories usually worked five full days per week, 11 hours per day, and a shorter working day on Saturdays.^72^ Because it is unclear how these shorter working days affected wages, the annual income of a male factory labourer is calculated based on five days per week, 260 days per year.^73^ For children's wages we turn to the 1890 labour surveys. Weavers’ assistants (mostly children) in Enschede earned on average 1.80 fl. per week when they were 12 years old, increasing to 2.35 fl. at the age of 13, and 3.50 fl. from the age of 14^74^ (a value of respectively 2.04 fl., 2.66 fl., and 3.96 fl. in 1910). The rise in wages was the result of the decreasing time children had to spend at the factory school.

The catalogue of the 1909 exhibition on home industry listed information about the labourer who made every object on display, their working hours, and how much they were paid.^75^ The textile and apparel industries were best represented, with objects produced in 390 different households. In total, the catalogue gave information about 144 men and 110 women who worked *alone* in the textile and apparel industry.^76^ On average, men earned 7.87 fl. per week for 76 hours of work and women earned 3.82 fl. per week for 57 hours of work. There were noteworthy differences in earnings between age groups. During their twenties and early thirties, the number of hours worked per day decreased from 10 to six and thereafter increased again.^77^

Table 3 summarizes the relevant information needed for the following section, in which the relative importance of all household resources will be calculated based on a fixed family size.

**Table 3:** Annual wage earnings of men, women, and children

	Agriculture	Industry
Husband	379.27	392.60
Wife	76.00	198.64
Child >12	37.00	205.92^a^

*Note: a* Wage for children aged 14 and older.

*Sources*: See footnotes to section IV.

## V

The final pieces of empirical evidence that need to be discussed relate to the costs of living. Many scholars agree that the commonly used ‘subsistence basket’ contains too few calories to survive. However, this basket is used here as a deflator of income to facilitate comparisons with earlier studies on Dutch living standards. Additionally, the price of a ‘respectability basket’, likewise constructed by Allen, has been calculated, which contains more calories and more luxurious foodstuffs (such as meat, butter, and cheese) and is a more realistic estimation of households’ consumptive needs.^78^ To determine the number of required baskets, age‐ and gender‐specific consuming unit equivalents set by the Food and Agricultural Organization are used. The daily caloric intake of the baseline consuming unit is set at 2,900 kcal.^79^

Table 4 presents the absolute and relative shares of income from land and men's, women's, and children's wage labour for both case‐study households with a fixed family size: a husband, a wife, and four children aged 6, 9, 11, and 14. This means that only one child was providing a wage income.^80^ The results show that while textile households generated 86 per cent of their total income from wage labour, this was only 55 per cent for agricultural households, where almost half of the income was derived from their own land.^81^ Still, in textile households too, the yields from land were a valuable addition to the household income, accounting for almost 14 per cent. Wage labour by women and children in agricultural households was of little significance since most of their labour time was spent on the family farm. In the textile households, women and children's wage incomes were more substantial, both constituting slightly over 20 per cent of household income.

**Table 4:** Household income composition and welfare ratios

Absolute and relative income					
		Agriculture		Textile	
		f	%	F	%
Self‐employed agriculture		401.92	44.9	125.55	13.6
Wage labour	*Husband*	*379.27*	*42.4*	*392.60*	*42.5*
	*Wife*	*76.00*	*8.5*	*198.64*	*21.5*
	*Children*	*37.00*	*4.1*	*205.92*	*22.3*
	Total	492.27	55.1	775.84	86.4
Total income		894.19 fl.		922.71 fl.	

Welfare ratios					
		Agriculture		Textile	
		Male breadwinner model	Total household income	Male breadwinner model	Total household income
Subsistence level		2.42	5.71	2.35	5.51
Respectability level		0.95	2.25	0.93	2.17

*Sources*: See footnotes to sections III and IV.

The households’ welfare ratios were well above subsistence level (table 4) even if the husband's wage had been the sole source of income. However, when using the respectability basket as a deflator, it appears that the annual wage income of the husband was not high enough. Interestingly, the welfare ratios of the two case‐study households were almost the same, even though their income composition differed considerably. This is in line with the notion that the agricultural and textile labour markets were tightly linked.^82^

These estimates can be validated by looking at other contemporary sources on household income, such as a sample of 25 rural household budgets from 1910. They corroborate that portfolios were diversified, although the exact distribution over the sources of income differed greatly between the households under investigation.^83^ All households generated income from both wage labour and land, ranging from 2 to 580 *are* with a median of 25.5 and a mean of 76.5 *are*. The husbands on average earned 354.34 fl. annually, which constituted 54 per cent of the total household income (with a range of 10 to 97 per cent). However, the average yields from land were lower than in our standardized household, with an average of 25 per cent of the total income, with a range of –1 to 80 per cent. This may have to do with the fact that not all households used their land to its fullest potential. Furthermore, a category of ‘divers’ incomes was included, such as inheritance or earnings from hosting a lodger, which further affected the income distribution.

**Figure 2:**
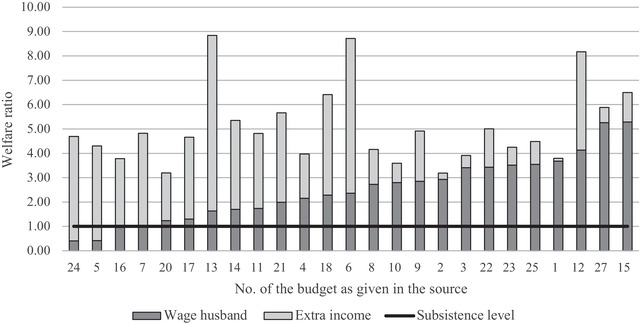
Welfare ratios of 25 agricultural households at subsistence level, *c*. 1910 *Notes and sources*: The numbers on the x‐axis refer to the individual household budgets. See online app. S7 for the underlying data. See online app. S7 for the same analysis at the respectability level.

For every household, the welfare ratio has been calculated based on the husband's wage alone and based on the total household income (figure 2). With the information about the number and age of the household members, the costs of living have been determined, taking regional price differences into account. Figure 2 reveals that all 25 households lived well above subsistence level. Furthermore, the average welfare ratios closely resemble those presented in table 4. At subsistence level, the average welfare ratio is 5.08 based on the total household income and 2.51 based solely on the husband's wage. At the respectability level it is 2.00 and 0.99 respectively.^84^ These results provide confidence that the results presented in table 4 are reliable estimate of the welfare ratios of the average rural household.

## VI

This section examines how household living standards changed throughout the life cycle, which starts in the year a man and a woman got married, and ends when the youngest child leaves the household at the age of 18. To calculate life cycle welfare ratios, we thus need to understand changes in income as well as consumption.

It is likely that the income from land was stable throughout the life cycle. Prior research has shown that in 1910, for the cultivation of one hectare of land, the labour input of 0.25 ‘man years’ was used.^85^ Although these calculations apply to large farms, they are indicative of the amount of time a wage labourer had to spend on his land. After all, wage labourers not only rented their land from their employers; they were often allowed to use ploughs and horses for soil preparation as well.^86^ The textile labourers in Twente did not have this equipment at their disposal, but their land was generally much smaller and could easily be cultivated on the side. Still, for both types of households the division of labour may have changed, with children taking over tasks from their parents once they became physically capable of doing so.

Husbands’ wage incomes are likely to have been stable too, since men were not affected physically by the arrival of children. This was obviously different for women who, besides bearing the physical burden of having children, were expected to carry out most of the care tasks. Indeed, wages in the textile home industry show clear life‐cycle changes, with earnings falling significantly between the ages of 20 and 30, rising thereafter, and starting to decrease again after the age of 40. These fluctuations were mainly the result of changing number of hours worked per day.^87^ For women working in agriculture, it is assumed that they stopped working for wages roughly three months before and half a year after a child was born. Finally, for older children working in the textile factories, wage differences between age groups (as explained in section IV) are taken into account, while stagnant wages are assumed for children working in agriculture. Finally, children younger than 12 did not work for wages at all.

To reconstruct changing consumption needs, we need to know how many and when children were born, and of which sex. To illustrate this, the life‐course of two Dutch families is used.^88^ The first household lived in Ferwerderadeel in the northern province of Friesland. The husband was an agricultural wage labourer with five children: three girls and two boys. The husband in the second household was a cotton weaver from Goor (Twente). This couple had six children, two of whom died shortly after they were born, leaving them with four children to raise and feed. Note that even though these households were not exceptional in terms of the number of children, they are meant to serve as *examples* of how household composition could develop, rather than representing the standard.^89^ Based on this information, the number of male consumer equivalents present in these households has been calculated for every single year of their life cycles.^90^ This number is multiplied by the costs of a subsistence consumption basket as well as a respectability consumption basket in the year 1910 to determine the total costs of living.^91^

**Figure 3:**
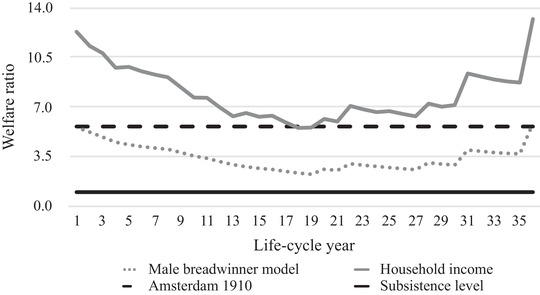
Life‐cycle welfare ratios (agriculture, subsistence) *Sources*: See footnotes to sections III, IV, and V.

**Figure 4:**
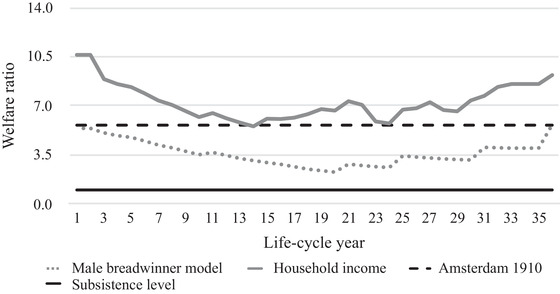
Life‐cycle welfare ratios (textiles, subsistence) *Sources*: See footnotes to sections III, IV, and V.

**Figure 5:**
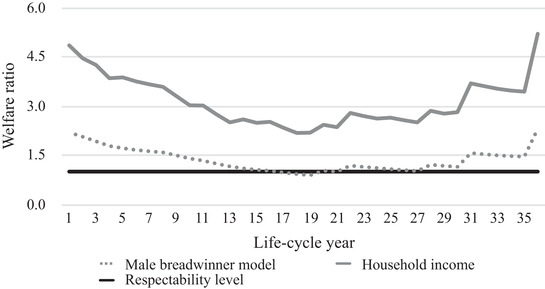
Life‐cycle welfare ratios (agriculture, respectability) *Sources*: See footnotes to sections III, IV, and V.

**Figure 6:**
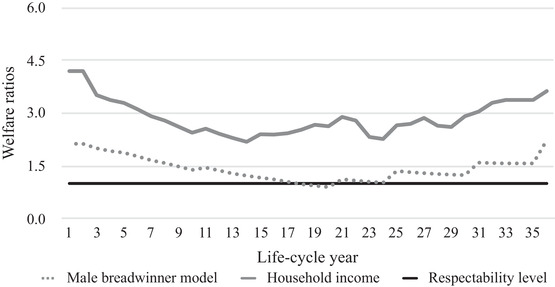
Life‐cycle welfare ratios (textiles, respectability) *Sources*: See footnotes to sections III, IV, and V.

Figures 3, 4, 5, 6 show the life‐cycle welfare ratios for both case‐study households at the subsistence level as well as the respectability level. Note that the exact trajectory of the welfare ratios was affected by the number of children and the point in the life cycle at which they were born. However, the general trends can be interpreted as representative for other agricultural and textile households. Crucially, two possible scenarios are presented: one based on the male breadwinner model, with the husband's wage as the sole income, and another based on the total household income. Figures 3 and 4 additionally include the subsistence welfare ratio in Amsterdam in the year 1910.^92^

This life‐cycle approach to living standards yields several interesting findings, validating the critique of the welfare ratio method discussed in section I. First, the differences between the male breadwinner model and the household income model are significant. For agricultural households, the incomes from land made up the largest share of the extra income, while in the textile households, women's wage labour was the most important extra resource, at least until the children were allowed to work in the factory. In particular, when two children earned wages, their relative income was significant and could amount to 30 per cent of the total household income.

Second, the differences between the two models is not constant, which means that the male breadwinner model misrepresents not only households’ living standards, but also their life‐cycle trajectory. Based on the male breadwinner model, households had the most difficult time after *c*. 20 years, because *all* children were present in the household, which means that the number of required consumption baskets was at its peak and, therefore, welfare ratios were at their lowest. The household income model, however, shows that the low point occurred earlier. In the textile households, welfare ratios started to increase again after 14 years, which was the point at which the oldest child was allowed to work in a factory and the household suddenly had a new source of income. This textile household experienced a second dip at 24 years, because the oldest children left the household and the wage earnings of the wife fell simultaneously.^93^ The situation was different in our agricultural household, where the trajectory of the household income model more closely resembled the male breadwinner model, because the income from land, the most important additional resource, was stable throughout the life cycle.

Third, following from the second point, because textile households only had limited access to subsistence resources, they were more vulnerable to shocks in the wider economy than agricultural households. Had we considered wage labour as the only indicator of households’ well‐being, we may have concluded that agricultural households were more vulnerable because rural labour demand was generally lower and seasonal, especially for women and children. Thus, this point supports the claim that the inclusion of access to land is crucial for understanding living standards and inequality within and between countries in the past.

Fourth, the results inform us about these households’ absolute level of material welfare. Both households would have been able to stay above subsistence level if the husband's wage had been the sole source of income. However, when we compare the results with the previously established welfare ratio for Amsterdam in 1910, it becomes clear that only during the very first and final years of the life cycle could our case‐study households have reached this level of welfare with one man's wage.^94^ Furthermore, figures 5 and 6 show that one man's annual wage in both agriculture and the textile industry could not have provided for the entire family at the respectability level during every life‐cycle stage. Since the respectability basket is a more realistic estimation of the actual costs of living, these figures leave little doubt that by the early 1900s, the male breadwinner model was *not* the dominant type of labour division in all working‐class households.

## VII

This article set out to develop a methodology for reconstructing historical living standards that accounts for diversified household portfolios and life‐cycle fluctuations. This methodology was applied to a case study of two types of Dutch households around the turn of the twentieth century. For this purpose, qualitative and quantitative information was collected on subsistence agriculture and gender‐ and age‐specific labour force participation and wage rates. By using household income as the numerator in calculating welfare ratios, as opposed to one man's wage, this study has provided a more thorough understanding of how households functioned. Furthermore, by considering life‐cycle changes in household income and consumption, it has revealed that living standards were dynamic.

The results show that both agricultural and textile households generated an important part of their income from cultivating land and keeping animals. The calculation based on a fixed family size has shown that the yields from land and livestock constituted 45 per cent of total household income in the former household and 14 per cent in the latter. Because the cultivation of these relatively small plots of land did not require full‐time labour input, it could, with the labour input of all household members, easily be combined with wage labour. The husband's wage labour was by far the most important resource in the textile households, followed by the wage labour of women and children. Men's wages in agricultural households were important too, while the wages of women and children were low. Thus, local labour market structures and demand for labour were the most important determinant of household portfolios.

The subsequent life‐cycle analysis has demonstrated that the annual wages of male agricultural and textile labourers were in theory high enough to sustain their families at the subsistence level, but not at the respectability level. Seeing that the respectability basket better reflects households’ consumptive needs, this finding underscores that men's wages are unlikely to have been the only source of income in working‐class households. Furthermore, the welfare ratios based on household income were not only structurally higher than those based on the male breadwinner model, but also followed a different life‐cycle trajectory.

These findings are important for at least two strands of literature. First, they reflect those of earlier studies that criticize conventional methods of researching historical living standards. Especially outside large urban centres, household income portfolios were diversified, with land and wage labour by all household members being important resources. Analyses based on men's real wages only sketch the rough outlines of how households functioned. Second, the present article has further expanded our understanding of Dutch female labour force participation. Based on both qualitative and quantitative sources and methods, it has supported the thesis that female labour force participation was more extensive than occupational censuses have long led us to believe. Thus, for large groups in society, the male breadwinner model was not the most common type of household labour division.

The scope of this study was limited in terms of period. Further research on the long‐term development of household income is needed to test whether or not men's real wages are a good reflection of *long‐term trends* in living standards. Furthermore, comparisons between different case studies will have to show whether previous findings about historical inequality based on men's urban real wages are upheld when household income is used instead as the numerator in calculating welfare ratios. Historically, access to subsistence resources, notably land, has been the most important determinant of living standards. Thus, a thorough understanding of how households’ access to land and wage labour has changed through time and space is a crucial missing piece of the puzzle for research on the long‐term development of global inequality. The methodology presented in this article can be applied to other case studies and can serve these future avenues of research.

## Supporting information

S1. Hectares of land cultivated by agricultural wage labourers
S2. Wages
S3. Absolute and relative number of male, female, and child labourers included in the 1889 survey on Dutch factories
S4. Results from the 1914 survey: marital status and age of home‐industrial workers and their distribution across sectors
S5. Costs of living
S6. Agricultural household budgets
S7. Historical Sample of the Netherlands: average number of children and child mortality
S8. Income and consumption during the life cycle

## Footnotes

^1^
Allen, ‘Great divergence’; Clark, ‘Farm wages’; Frankema and van Waijenburg, ‘Structural impediments’; Allen, Bassino, Ma, Moll‐Murata, and van Zanden, ‘Wages, prices and living standards’; Arroyo Abad, Davies, and van Zanden, ‘Between conquest and independence’; de Zwart, Globalization; de Zwart and van Zanden, ‘Labor, wages, and living standards’.

^2^
de Haas, ‘Measuring rural welfare’; Schneider, ‘Real wages’.

^3^
Pott‐Buter, *Facts and fairy tales*.

^4^
Schmidt and van Nederveen Meerkerk, ‘Reconsidering’; Boter, ‘Ideal versus reality?’.

^5^
van Zanden, ‘Wages and the standard of living’, p. 178.

^6^
Phelps Brown and Hopkins, ‘Seven centuries’; eisdem, *Perspective*; van Zanden, ‘Wages and the standard of living’.

^7^ For an overview, see Humphries, ‘Lure of aggregates’.

^8^
Horrell and Humphries, ‘Women's labour force participation’.

^9^
Horrell, Meredith, and Oxley, ‘Measuring misery’.

^10^ Borderías, Pérez‐Fuentes, and Sarasúa, ‘Gender inequalities’, p. 191.

^11^ Schneider, ‘Real wages’.

^12^
de Haas, ‘Measuring rural welfare’.

^13^ Horrell and Humphries, ‘Women's labour force participation’, p. 105.

^14^ Schneider, ‘Real wages’, p. 103. In his thesis Schneider (‘Studies’) constructs a model that includes women's and children's earnings. This work has, to my knowledge, not yet been published.

^15^
van Zanden, *De economische ontwikkeling*, pp. 332–6; Bieleman, *Boeren in Nederland*, pp. 280–1.

^16^
Hendrickx, ‘From weavers to workers’.

^17^
van Nederveen Meerkerk, ‘Challenging’.

^18^
Hendrickx, ‘From weavers to workers’, pp. 333–6.

^19^ The term ‘protoindustrialization’ was coined by Franklin Mendels in the 1970s and scholars have debated the applicability of this theory ever since; Mendels, ‘Proto‐industrialization’. An in‐depth discussion of this debate falls outside the scope of this article.

^20^
Pott‐Buter, *Facts and fairy tales*, p. 21.

^21^
Schmidt and van Nederveen Meerkerk, ‘Reconsidering’.

^22^
Higgs, ‘Women, occupations and work’; Hill, ‘Women, work and the census’; Humphries and Sarasua, ‘Off the record’; van Nederveen Meerkerk and Paping, ‘Beyond the census’; Muñoz Abeledo, ‘Women in the rural and industrial labor force’; Sarasua, ‘Women's work and structural change’.

^23^
Schmidt and van Nederveen Meerkerk, ‘Reconsidering’; Boter and Woltjer, ‘Impact of sectoral shifts’; Boter, ‘Dutch divergence?’.

^24^ This law limited women's and children's working days to 11 hours and prohibited their labour at night and on Sundays. Furthermore, women were not allowed to work within four weeks after giving birth.

^25^ In other European countries, such as Germany and the UK, similar exhibitions had been created during the early 1900s. The Netherlands were thus part of an international trend of gaining insight into the working and living conditions of the labouring class. Directie van den Arbeid, *Onderzoekingen*, pt. I, pp. 2–29.

^26^
Directie van den Arbeid, *Onderzoekingen*, pt. I; idem, *Onderzoekingen*, pt. II; idem, *Onderzoekingen*, pt. III; Posthumus, *Huisindustrie in Nederland*.

^27^
Staatscommissie voor den Landbouw, *Algemeen overzicht*; idem, *Groningen‐Gelderland*; idem, *Utrecht‐Limburg*; idem, *Rapporten (landarbeiders)*.

^28^ Staatscommissie voor den Landbouw, *Overzicht van het landbouwbedrijf*, p. 417. The exact way in which agricultural households generated income from their land differed per region and household. Some used their land for keeping cows and sold their milk to dairy factories. Others used their land for the cultivation of potatoes and vegetables, or combined the two. To avoid overcomplicating the analysis, I chose to calculate the value of land when used for potato cultivation. Goats and chickens were commonly held for households’ own consumption.

^29^
van Zanden, *De economische ontwikkeling*, p. 274.

^30^
Zwemer, *Een zekel*, p. 22.

^31^
Staatscommissie voor den Landbouw, *Overzicht van het landbouwbedrijf*, p. 246. See also ibid., pp. 339, 412; idem, *Schetsen van het landbouwbedrijf*, p. 354; idem, *Rapporten*, p. 137.

^32^
Arbeidsinspectie, *Twente*, p. 132.

^33^ Hendrickx, ‘Een wereld van verschil?’; Mastboom, ‘Protoindustrialization and agriculture’.

^34^ Hendrickx, ‘From weavers to workers’, p. 350.

^35^
Heerma van Voss, ‘Twente 1920’; Hendrickx, ‘Family, farm and factory’.

^36^ Because several types of square measures were used in the sources, I converted everything into *ares* and hectares. ‐ 1 hectare = 100 × 100 metres = 10,000 m^2^
‐ 1 hectare = 100 *are*
‐ 1 hectare = 700 *roede*
‐ 1 *are* = 10 × 10 metres = 100 m^2^ = 0.01 hectare

^37^ This is the weighted average of all land being rented by agricultural wage labourers. Another much smaller group of wage labourers owned land. See online app. S1, tab. S1.1.

^38^
van Riel, ‘Trials of convergence’, p. 705.

^39^ Staatscommissie voor den Landbouw, *Algemeen overzicht*, pp. 85–96; Koninklijke Nederlandsche Landbouwvereeniging, *Bijzondere catalogus*, p. 52.

^40^ Cited in Bieleman, *Boeren op het Drentse zand*, p. 512.

^41^ Ibid., pp. 510–16.

^42^
Arbeidsinspectie, *Verslag van de tweede afdeling*, p. 22. Note that the exact measurements of one ‘spint’ are unclear and differed between regions. From this quotation, it seems that it was about 225 m^2^ which equals 2.25 are.

^43^ Arbeidsinspectie, *Twente*, pp. 240, 249. An analysis of the 1914 survey on home industry yields similar results. In total, 3,813 households were included that cultivated land. The weighted average of the size of the land was 16.2 are, which is slightly skewed by some 100 households cultivating between 200 and 1000 are of land. Excluding those resulted in an average of a little more than 10 are per household. Note that these were mostly households living in North Brabant.

^44^
Arbeidsinspectie, *Verslag van de tweede afdeling*, p. 23.

^45^ Directie van den Arbeid, *Onderzoekingen*, pt. I; idem, *Onderzoekingen*, pt. II; idem, *Onderzoekingen*, pt. III. In 1881, a goat cost 6.50 fl. and a chicken 0.77 fl. in the province of Gelderland; Gedeputeerde Staten, *Verslag van den toestand der provincie Gelderland*, p. 887.

^46^
Departement van Landbouw Nijverheid en Handel, *Verslag*, p. 51. Note that how many potatoes could be cultivated per hectare of land depended on the type of soil and therefore varied from region to region. The numbers used here are the national average.

^47^
de Haas, ‘Measuring rural welfare’, pp. 12–13.

^48^ See, for instance, de Janvry, Fafchamps, and Sadoulet, ‘Peasant household behaviour’, pp. 1401–2.

^49^ Potato starch was used for glue and as a binder in the food industry, among other things.

^50^
Sociaal Democratische Studieclub, *Landarbeiders*, pp. 46–56.

^51^ van Nederveen Meerkerk and Paping, ‘Beyond the census’, p. 461.

^52^
Paping, *‘Voor een handvol stuivers’*, pp. 332–3; van Nederveen Meerkerk and Paping, ‘Beyond the census’, p. 459.

^53^ Staatscommissie voor den Landbouw, *Algemeen overzicht*, p. 240.

^54^
Priester, *De economische ontwikkeling*, p. 174.

^55^
van Zanden, *De economische ontwikkeling*, p. 78.

^56^ Sociaal Democratische Studieclub, *Landarbeiders*, pp. 114–16.

^57^ Ibid., p. 108.

^58^ Boter, ‘Ideal versus reality?’, p. 92.

^59^
Schenkeveld, *Het Kinderwetje*. Note that this law did not apply to children working in agriculture.

^60^
Arbeidsinspectie, *Verslag van de derde afdeling*, p. 261.

^61^ G. Reudink, ‘Nijverheidsstatistiek Struve en Bekaar’ (1994), http://www.neha.nl/struve/ (accessed May 2019). Note that only factories or workshops in which more than 10 people worked were included in this investigation. This census is fundamentally different from the regular occupational censuses, for which the census takers visited households, asked each household member about their occupation, and then decided for themselves whether or not they would register this person with an occupation.

^62^ See online app. S3.

^63^
Departement van Landbouw Nijverheid en Handel, *Onderzoek naar den fabrieksarbeid*.

^64^ Arbeidsinspectie, *Twente*, p. 378.

^65^
Arbeidsinspectie, *Verslag van de derde afdeling*. Also quoted in Boter, ‘Ideal versus reality?’, p. 91.

^66^ See online app. S4 for more information about the distribution of all homeworkers across different sectors.

^67^ Around 1900, Dutch women's average age at first marriage was between 25 and 28 years; van Poppel, *Trouwen in Nederland*, p. 142.

^68^ See online app. S5, which additionally reports the wages of male farmers, and male and female servants. Source: Koninklijke Nederlandsche Landbouwvereeniging, *Bijzondere catalogus*, pp. 36–50.

^69^ Sociaal Democratische Studieclub, *Landarbeiders*, pp. 102–3; Staatscommissie voor den Landbouw, *Algemeen overzicht*.

^70^ Sociaal Democratische Studieclub, *Landarbeiders*, pp. 108–13 (quotation p. 109).

^71^ See online app. S2. Wages varied between occupations and regions. A male weaver on average earned 1.45 fl. per day and a female weaver 1.08 fl. The gender wage gap for spinners was much larger with male spinners earning 2.10 fl. and female spinners 1.20 fl. per day. Furthermore, it should be noted that textile wages in Twente were relatively high. In Noord Brabant, men's day wages in cotton textile manufacturing were on average 1.27 fl. and women's day wages 0.72 fl., and in the Achterhoek 1.05 fl. and 0.72 fl. respectively.

^72^
Humphries and Weisdorf, ‘Wages of women’.

^73^ For a discussion of the length of a typical working year, see also ibid.

^74^
Arbeidsinspectie, *Verslag van de eerste afdeling*, p. 13.

^75^
Posthumus, *Huisindustrie in Nederland*, pp. 46–79.

^76^ In some of those households, there was only one labourer responsible for the displayed objects, but in others two or more people worked together. In the latter case, it was impossible to extract individual wage earnings.

^77^ See online app. S2 for more information about the earnings per age group.

^78^ See online app. S5 for more information about the construction of the consumption baskets, including information on regional price differences.

^79^ FAO, *Human energy requirements*. See online app. S5, tab. S5.4, for all gender‐ and age‐specific consuming unit equivalents. Note that the respectability basket contains 2,900 kcal and is thus in that sense equal to the baseline consuming unit. These calculations have also been used by other historians: Humphries, ‘Lure of aggregates’; Schneider, ‘Real wages’.

^80^ For the price of annual consumption baskets I have used the underlying data for Amsterdam in Allen et al., ‘Wages, prices and living standards’, p. 23 (including rent by adding 5% of the price of one basket). To convert prices in grams of silver into prices in guilders I have used Allen's online dataset: R. C. Allen, ‘Amsterdam’ https://www.nuffield.ox.ac.uk/people/sites/allen-research-pages/ (accessed on 24 July 2019).

^81^ Note that I assume that these households used every piece of land and that the harvest was optimal.

^82^ Hendrickx, ‘Family, farm and factory’.

^83^ See online app. S6 for the underlying data and more information about the calculations.

^84^ Note that the contribution of men's wages was not necessarily based on full‐time employment.

^85^
van Zanden, *De economische ontwikkeling*, p. 133.

^86^
Zwemer, *Een zekel*, p. 22.

^87^ See online app. S2.

^88^ I used the HSN (Historical Sample of the Netherlands) database, which contains information about the life courses of 85,500 Dutch individuals born between 1812 and 1922; Historical Sample of the Netherlands (HSN), ‘Data set life courses’, release 2010.01 (n = 37.137), version: Historical Sample of the Netherlands as linked data, https://druid.datalegend.net/HSN/HSN (accessed Sept. 2019). For more information, see International Institute of Social History, ‘The Historical Sample of the Netherlands’, https://iisg.amsterdam/en/hsn (accessed Sept. 2019).

^89^ See online app. S7 for the summary statistics of 648 families included in the HSN database.

^90^ See online apps. S5 and 8.

^91^ Note that I corrected for regional price differences; see online app. S5.2.

^92^ Allen et al., ‘Wages, prices and living standards’, p. 27.

^93^ See section IV and online app. S2.

^94^ Note that I have not corrected for the effects of men's declining physical strength with age on their wage earnings. Further research will have to be done on this specific stage of the life cycle.
