# Supplementary material for: Living standards and the life cycle: reconstructing household income and consumption in the early twentieth‐century Netherlands
Source: Econ Hist Rev. 2020 Jun 16;73(4):1050–73. doi: 10.1111/ehr.12997 (PMC7687131; doi:10.1111/ehr.12997)
Supplement: Supplementary file 1 — S1. Hectares of land cultivated by agricultural wage labourers S2. Wages S3. Absolute and relative number of male, female, and child labourers included in the 1889 survey on Dutch factories S4. Results from the 1914 survey: marital status and age of home‐industrial workers and their distribution across sectors S5. Costs of living S6. Agricultural household budgets S7. Historical Sample of the Netherlands: average number of children and child mortality S8. Income and consumption during the life cycle [file EHR-73-1050-s001.docx]

**Living standards and the life-cycle: Reconstructing household income and consumption in the early-twentieth century Netherlands**

**Appendix S1.** **Hectares of land cultivated by agricultural wage labourers**

*Land*

Table S1.1 below shows the absolute and relative number of agricultural wage labourers that owned or rented a plot of land in one of seven ranges. The survey conductors included every person who performed wage labour in the agricultural sector, even if it was not their primary source of income. It remains unclear whether the investigators included female wage labourers individually or reported the amount of land per household. Since by 1913 the number of female wage labourers had drastically decreased relative to the nineteenth century and children were unlikely to own land, I assume the outcomes show the available land per household.

| Table S1.1 Hectares of land per province and per head cultivated by agricultural wage labourers, 1913 |
| --- |
| \| **Hectares of land** \| **Owners** \| \| **Renters** \| \| **Total** \| \| \| --- \| --- \| --- \| --- \| --- \| --- \| --- \| \|  \| *N* \| *%* \| *N* \| *%* \| *N* \| *%* \| \| 0.05-0.15 \| 2,230 \| 2.6% \| 13,850 \| 16.1% \| 16,080 \| 18.7% \| \| 0.15-0.25 \| 1,750 \| 2.0% \| 8,270 \| 9.6% \| 10,020 \| 11.6% \| \| 0.25-0.50 \| 4,370 \| 5.1% \| 12,084 \| 14.0% \| 16,454 \| 19.1% \| \| 0.50-1.00 \| 7,320 \| 8.5% \| 12,170 \| 14.1% \| 19,490 \| 22.6% \| \| 1.00-2.00 \| 6,580 \| 7.6% \| 8,790 \| 10.2% \| 15,370 \| 17.8% \| \| 2.00-3.00 \| 2,760 \| 3.2% \| 3,020 \| 3.5% \| 5,780 \| 6.7% \| \| 3.00-5.00 \| 1,360 \| 1.6% \| 1,560 \| 1.8% \| 2,920 \| 3.4% \| \| ***Total*** \| ***26,370*** \| ***30.6%*** \| ***59,744*** \| ***69.4%*** \| ***86,114*** \| ***100.0%*** \| |
| Sources: Departement van Landbouw Nijverheid en Handel, *Verslag over 1911;* Koninklijke Nederlandsche Landbouwvereeniging, *Bijzondere catalogus*, p. 54. |

*Number of livestock*

For information about agricultural households’ livestock I turned to the secondary literature. Goats were considered to be ‘the dairy cow of the poor’^[[1]](#footnote-1)^ because they provided milk – mainly for subsistence use − and did not require expensive forage as they were supposedly happy to consume the food remains from the kitchen. Furthermore, their excrements could be used as manure for the cultivation of land. Jan Bieleman has shown that 71% of all the wage labourers in Drenthe owned one or more goats (on average 1.7) as opposed to 27% of the independent farmers (on average 1.3).^[[2]](#footnote-2)^ Furthermore, he has estimated that almost 75% of the wage labourers in Drenthe owned chickens: on average 12 per household.^[[3]](#footnote-3)^ One chicken gave approximately 177 eggs per year which means that the average wage labourer’s household could consume and/or sell 2,124 eggs annually.^[[4]](#footnote-4)^

The survey on home industry from 1914 provided information about the livestock of 340 households in which at least one person was working in the home industry. In total, they kept 279 cows, 21 horses, and 383 pigs, sheep, or goats (on average 0.82, 0.06, and 1.13 per household respectively). By far most households included in the sample (40%) owned one pig, sheep, or goat. This source explicitly excluded information about chickens.^[[5]](#footnote-5)^

**Appendix S2. Wages**

*Agriculture*

Table S2.1 reports the annual earnings of agricultural wage labourers during the first decade of the twentieth century. These wages included the value of in-kind payments (except for the costs of food and shelter for the live-in servants), excluded the earnings of other household members, and accounted for seasonal unemployment.

| Table S2.1. Annual earnings in agriculture in guilders, ca. 1910^a^ |
| --- |
| \| **Province** \| **Farmer** \| **Wage labourer** \| **Male servant** \| **Female servant** \| \| --- \| --- \| --- \| --- \| --- \| \| Groningen \| 399.46 \| 372.87 \| 195.33 \| 106.48 \| \| Friesland \| 465.25 \| 417.92 \| 248.43 \| 129.85 \| \| Drenthe \| 330.00 \| 325.00 \| 178.33 \| 86.25 \| \| Overijssel \| 401.86 \| 326.67 \| 197.74 \| 110.10 \| \| Gelderland \| 352.25 \| 362.33 \| 172.87 \| 118.81 \| \| Utrecht \| 405.33 \| 375.00 \| 210.00 \| 173.67 \| \| Noord Holland \| 561.20 \| 537.70 \| 255.80 \| 140.04 \| \| Zuid Holland \| 492.48 \| 340.51 \| 237.48 \| 152.34 \| \| Zeeland \| 400.00 \| 368.00 \| 203.45 \| 102.63 \| \| Noord Brabant \| 405.50 \| - \| 203.33 \| 114.92 \| \| Limburg \| 455.00 \| 366.70 \| 219.00 \| 187.75 \| \| ***Average country*** \| ***424.39*** \| ***379.27*** \| ***211.07*** \| ***129.35*** \| |
| ^a^ Unweighted averages of municipality-level averages  **Sources**: Koninklijke Nederlandsche Landbouwvereeniging, *Bijzondere catalogus*, pp. 36-50. |

*Textile industry (factory)*

Table S2.2 reports the wages of men and women with various occupations in the textile industry in Twente in 1908.

| Table S2.2. Men’s and women’s day wages in the textile factories of Twente in guilders, 1908 |
| --- |
| \|  \| **Occupation** \| **Men** \| \| **Women** \| \| \| --- \| --- \| --- \| --- \| --- \| --- \| \|  \|  \| *Day wage (f)* \| *N* \| *Day wage (f)* \| *N* \| \| Spinning mill \| *Opsteker* (spinner’s assistant) \| 0.90 \| 129 \| 0.75 \| 199 \| \| Spinner \| 2.10 \| 173 \| 1.20 \| 120 \| \| Fibre lapper \| 1.48 \| 269 \| - \| 0 \| \| Winder \| - \| 0 \| 1.09 \| 348 \| \| Weaving mill \| *Spoelster* \| - \| 0 \| 0.96 \| 2,263 \| \| Shearer \| 1.59 \| 89 \| 1.36 \| 381 \| \| Fuller \| 1.94 \| 183 \| - \| 0 \| \| *Aandraaier* (weaver’s assistant) \| 1.43 \| 76 \| 1.09 \| 455 \| \| Weaver \| 1.45 \| 8,389 \| 1.08 \| 2,490 \| \| Supervisor \| 2.12 \| 231 \| - \| 0 \| \| Dye house and bleachery \| Dyer \| 1.71 \| 412 \| - \| 0 \| \| Bleacher \| 1.33 \| 623 \| 1.06 \| 86 \| \| Other \| Labourer in the storehouse \| 1.44 \| 490 \| 1.03 \| 272 \| \| Packer \| 1.50 \| 359 \| 1.20 \| 64 \| \| Carrier \| 1.40 \| 438 \| - \| 0 \| |
| Source: Centraal Bureau voor de Statistiek, *Statistiek (textiel industrie)*. |

*Textile and apparel industry (home industry)*

The most important source for home industrial earnings is the catalogue of the exhibition on the home industry dating from 1909. The catalogue listed information about the labourer who made every object on display, their working hours, and how much they were paid. For the present paper, I used all the objects within the textile, apparel, and shoe industries. It has to be noted that a large share of the objects were manufactured in households in which multiple people were working in the home industry. In these cases, it was impossible to determine individual wages.

The annual earnings of women working in the home industry depended on their age and thus the stage of their life-cycle. Table S2.3 below shows the weekly wages of women who worked alone in several age groups. The two right-most columns additionally give the average number of hours worked per day per woman. Unlike the wages, the length of the working day of individuals was listed in the catalogue, even if multiple people were active in the same household. Clearly, the number of hours worked per day decreased during the early stages of the life-cycle, when there were (probably) small children present in the household, which resulted in lower weekly and annual earnings.

| Table S2.3. Women’s wages in the home industry (textiles and shoes) |
| --- |
| \|  \| **Only women working alone** \| \| \| **All women** \| \| \| --- \| --- \| --- \| --- \| --- \| --- \| \| *Age group* \| *Weekly wage* \| *Annual wage ^a^* \| *N* \| *Hours worked per day* \| *N* \| \| 20-24 \| 4.92 \| 255.78 \| 24 \| 10 \| 51 \| \| 25-29 \| 3.29 \| 170.95 \| 16 \| 8 \| 32 \| \| 30-34 \| 3.35 \| 174.09 \| 14 \| 6 \| 32 \| \| 35-39 \| 3.54 \| 184.23 \| 7 \| 8 \| 20 \| \| 40-44 \| 5.54 \| 287.91 \| 15 \| 9 \| 24 \| \| 45-49 \| 2.78 \| 144.56 \| 5 \| 9 \| 18 \| \| 50-54 \| 1.80 \| 93.60 \| 7 \| 7 \| 17 \| \| 55-59 \| 2.30 \| 119.60 \| 5 \| 8 \| 9 \| \| 60-64 \| 2.19 \| 113.67 \| 5 \| 7 \| 12 \| \| 65-69 \| 2.75 \| 143.00 \| 3 \| 5 \| 12 \| |
| ^a^ Weekly wage times 52.  Source: Posthumus, *Huisindustrie in Nederland*. |

| Figure S2.1. Life-cycle changes in women’s home industrial wages in guilders |
| --- |
|  |
|  |

**Appendix S3.** **Absolute and relative number of male, female, and child labourers included in the 1889 survey on Dutch factories**

| Table S3.1. Absolute number of labourers per sector, 1889 |
| --- |
| \|  \| **Men** \| **Women** \| \| **Children (12-18)** \| \| **Total** \| \| --- \| --- \| --- \| --- \| --- \| --- \| --- \| \| **Nijverheidsgroep** \|  \| *Married* \| *Unmarried* \| *Boys* \| *Girls* \|  \| \| Pottery-, glass-, chalk- and stone production \| 10,180 \| 505 \| 313 \| 4,337 \| 453 \| 15,788 \| \| Metal processing \| 5,187 \| 11 \| 79 \| 1,036 \| 107 \| 6,420 \| \| Book- and lithography printing, printing of wood-, copper- and steel engraving \| 4,431 \| 4 \| 39 \| 2,039 \| 15 \| 6,528 \| \| Construction \| 1,485 \| 2 \| 42 \| 177 \| 1 \| 1,707 \| \| Chemical industry \| 1,550 \| 22 \| 161 \| 295 \| 245 \| 2,273 \| \| Wood-, cork, straw processing \| 5,824 \| 18 \| 85 \| 1,382 \| 187 \| 7,496 \| \| Apparel industry \| 1,837 \| 417 \| 1,388 \| 185 \| 734 \| 4,561 \| \| Art industry \| 135 \| 0 \| 0 \| 26 \| 0 \| 161 \| \| Leather, wax cloth and caoutchouc \| 535 \| 0 \| 0 \| 61 \| 1 \| 597 \| \| Paper production \| 1,206 \| 94 \| 144 \| 361 \| 229 \| 2,034 \| \| Shipbuilding and carriage production \| 6,787 \| 4 \| 11 \| 954 \| 5 \| 7,761 \| \| Steam- and other machinery building \| 7,658 \| 13 \| 29 \| 1,100 \| 24 \| 8,824 \| \| Textile industry \| 13,299 \| 1,116 \| 3,647 \| 4,205 \| 3,530 \| 25,797 \| \| Peat industry \| 25 \| 3 \| 6 \| 14 \| 2 \| 50 \| \| Lighting, oil, varnish, fat, etc. \| 4,001 \| 45 \| 318 \| 222 \| 336 \| 4,922 \| \| Food and tobacco industry \| 21,048 \| 422 \| 1,581 \| 5,378 \| 1,080 \| 29,509 \| \| Extraction of metals, peat, salt, etc. \| 433 \| 2 \| 4 \| 68 \| 0 \| 507 \| \| *Total* \| *85,621* \| *2,678* \| *7,847* \| *21,840* \| *6,949* \| *124,935* \| |
| Source: Reudink, *Nijverheidsstatistiek*. |

| Table S3.2. Share of male, female, and child labourers per sector, 1889 |
| --- |
| \|  \| **Men** \| **Women** \| \| **Children (12-18)** \| \| \| --- \| --- \| --- \| --- \| --- \| --- \| \| **Nijverheidsgroep** \|  \| *Married* \| *Unmarried* \| *Boys* \| *Girls* \| \| Pottery-, glass-, chalk- and stone production \| 64.5% \| 3.2% \| 2.0% \| 27.5% \| 2.9% \| \| Metal processing \| 80.8% \| 0.2% \| 1.2% \| 16.1% \| 1.7% \| \| Book- and lithography printing, printing of wood-, copper- and steel engraving \| 67.9% \| 0.1% \| 0.6% \| 31.2% \| 0.2% \| \| Construction \| 87.0% \| 0.1% \| 2.5% \| 10.4% \| 0.1% \| \| Chemical industry \| 68.2% \| 1.0% \| 7.1% \| 13.0% \| 10.8% \| \| Wood-, cork, straw processing \| 77.7% \| 0.2% \| 1.1% \| 18.4% \| 2.5% \| \| Apparel industry \| 40.3% \| 9.1% \| 30.4% \| 4.1% \| 16.1% \| \| Art industry \| 83.9% \| 0.0% \| 0.0% \| 16.1% \| 0.0% \| \| Leather, wax cloth and caoutchouc \| 89.6% \| 0.0% \| 0.0% \| 10.2% \| 0.2% \| \| Paper production \| 59.3% \| 4.6% \| 7.1% \| 17.7% \| 11.3% \| \| Shipbuilding and carriage production \| 87.5% \| 0.1% \| 0.1% \| 12.3% \| 0.1% \| \| Steam- and other machinery building \| 86.8% \| 0.1% \| 0.3% \| 12.5% \| 0.3% \| \| Textile industry \| 51.6% \| 4.3% \| 14.1% \| 16.3% \| 13.7% \| \| Peat industry \| 50.0% \| 6.0% \| 12.0% \| 28.0% \| 4.0% \| \| Lighting, oil, varnish, fat, etc. \| 81.3% \| 0.9% \| 6.5% \| 4.5% \| 6.8% \| \| Food and tobacco industry \| 71.3% \| 1.4% \| 5.4% \| 18.2% \| 3.7% \| \| Extraction of metals, peat, salt, etc. \| 85.4% \| 0.4% \| 0.8% \| 13.4% \| 0.0% \| |
| Source: Reudink, *Nijverheidsstatistiek*. |

**Appendix S4.** **Results from the 1914 survey: Marital status and age of home-industrial workers and their distribution across sectors**

| Table S4.1. Number of home industrial workers by marital status, 1914 |
| --- |
| \| **Marital status** \| **Men** \| \| **Women** \| \| **Unknown gender** \| \| **Total** \| \| \| --- \| --- \| --- \| --- \| --- \| --- \| --- \| --- \| --- \| \|  \| *n* \| *%* \| *n* \| *%* \| *n* \| *%* \| *n* \| *%* \| \| Married \| 3,423 \| 25.4 \| 4,425 \| 39.6 \| 0 \| 0.0 \| 7,848 \| 31.4 \| \| Unmarried^a^ \| 2,908 \| 21.6 \| 3,669 \| 32.8 \| 318 \| 100.0 \| 6,895 \| 27.6 \| \| Widow(er)^b^ \| 11 \| 0.1 \| 739 \| 6.6 \| 0 \| 0.0 \| 750 \| 3.0 \| \| Unknown \| 7,142 \| 53.0 \| 2,352 \| 21.0 \| 0 \| 0.0 \| 9,494 \| 38.0 \| \| ***Total*** \| ***13,484*** \| ***100.0*** \| ***11,185*** \| ***100.0*** \| ***318*** \| ***100.0*** \| ***24,987*** \| ***100.0*** \| |
| ^a^ Including children.  ^b^ The survey conductors did normally not have a separate category for widowers, only for widows.  Sources: Directie van den Arbeid, *Onderzoekingen (part I, II, and III)*. |

| Table S4.2. Number of home industrial workers by age, 1914 |
| --- |
| \| **Age** \| **Men** \| \| **Women** \| \| **Unknown gender** \| \| **Total** \| \| \| --- \| --- \| --- \| --- \| --- \| --- \| --- \| --- \| --- \| \|  \| *n* \| *%* \| *n* \| *%* \| *n* \| *%* \| *n* \| *%* \| \| <12 \| 790 \| 5.9 \| 912 \| 8.2 \| 179 \| 70.8 \| 1,881 \| 7.6 \| \| 12-15 \| 802 \| 6.0 \| 1,061 \| 9.6 \| 74 \| 29.2 \| 1,937 \| 7.9 \| \| 16-19 \| 919 \| 6.9 \| 1,069 \| 9.7 \| 0 \| 0.0 \| 1,988 \| 8.1 \| \| 20-24 \| 352 \| 2.6 \| 538 \| 4.9 \| 0 \| 0.0 \| 890 \| 3.6 \| \| 25-29 \| 2,038 \| 15.3 \| 1,646 \| 14.9 \| 0 \| 0.0 \| 3,684 \| 15.0 \| \| 30-39 \| 2,844 \| 21.4 \| 2,101 \| 19.0 \| 0 \| 0.0 \| 4,945 \| 20.1 \| \| 40-49 \| 2,254 \| 16.9 \| 1,529 \| 13.8 \| 0 \| 0.0 \| 3,783 \| 15.4 \| \| 50-59 \| 1,569 \| 11.8 \| 802 \| 7.2 \| 0 \| 0.0 \| 2,371 \| 9.6 \| \| 60-64 \| 211 \| 1.6 \| 171 \| 1.5 \| 0 \| 0.0 \| 382 \| 1.6 \| \| 65-69 \| 775 \| 5.8 \| 346 \| 3.1 \| 0 \| 0.0 \| 1,121 \| 4.6 \| \| >70 \| 419 \| 3.1 \| 240 \| 2.2 \| 0 \| 0.0 \| 659 \| 2.7 \| \| Unknown \| 333 \| 2.5 \| 657 \| 5.9 \| 0 \| 0.0 \| 990 \| 4.0 \| \| ***Total*** \| ***13,306*** \| ***100.0%*** \| ***11,072*** \| ***100.0%*** \| ***253*** \| ***100.0%*** \| ***24,631*** \| ***100.0*** \| |
| Sources: Directie van den Arbeid, *Onderzoekingen (part I, II, and III)*. |

| Table S4.3. Number of home industrial workers per sector, 1914 ^a^ |
| --- |
| \| **Industry** \| **Men** \| \| **Women** \| \| **Total** \| \| \| --- \| --- \| --- \| --- \| --- \| --- \| --- \| \|  \| *n* \| *%* \| *n* \| *%* \| *n* \| *%* \| \| Food and tobacco industry \| 3,184 \| 23.6 \| 6,383 \| 57.1 \| 9,885 \| 39.6 \| \| Textile and apparel industry \| 5,600 \| 41.5 \| 3,279 \| 29.3 \| 8,879 \| 35.5 \| \| Wood-, cork, straw processing \| 1,562 \| 11.6 \| 755 \| 6.8 \| 2,317 \| 9.3 \| \| Leather, wax cloth and caoutchouc \| 2,546 \| 18.9 \| 163 \| 1.5 \| 2,709 \| 10.8 \| \| Other \| 592 \| 4.4 \| 605 \| 5.4 \| 1,197 \| 4.8 \| \| ***Total*** \| ***13,484*** \| ***100*** \| ***11,185*** \| ***100*** \| ***24,987*** \| ***100*** \| |
| ^a^ In the food and tobacco industry, the gender of 318 workers was unknown, making the total number of workers in this industry higher than the sum of the male and the female workers included in the table.  Sources: Directie van den Arbeid, *Onderzoekingen (part I, II, and III)*. |

**Appendix S5.** **Costs of living**

*Constructing a Dutch respectability basket*

It is widely acknowledged that the contents of the subsistence basket as developed by Robert Allen contain insufficient calories. Recently, more realistic estimations have been made based on the *Human Energy Requirements* report by the *Food and Agricultural Organization*. Furthermore, besides the subsistence baskets, Allen composed a respectability basket, which contains more calories. For this paper, I have used the contents of Allen’s respectability basket (Table S5.1). To calculate the annual costs of each good, I used the price series composed by Arthur van Riel.^[[6]](#footnote-6)^

Two things need to be specified. First, van Riel only gives index numbers for the prices of linen. Therefore, I used the silver price Allen gives for Amsterdam in 1799, I assumed the price was equal to the year 1818, and then used van Riel’s index numbers to calculate the price in 1910. Second, I changed the amount of bread from 234 kilograms to 294 kilograms per year to match the caloric contents to the baseline set by the FAO (see section 5.3 in this appendix).

| Table S5.1. Respectability basket 1910 |
| --- |
| \| **Good** \| **Specification of good** \| **Quantity per consuming unit per year** \| **Price in guilders** \| \| --- \| --- \| --- \| --- \| \| Bread \| Ryebread \| 294 kg ^a^ \| 26.46 \| \| Beans/peas \| Peas \| 52 L \| 6.86 \| \| Meat \| Beef \| 26 kg \| 19.11 \| \| Butter \| - \| 5.2 kg \| 7.10 \| \| Cheese \| - \| 5.2 kg \| 2.97 \| \| Eggs \| - \| 52 each \| 2.31 \| \| Beer \| - \| 182 L \| 7.28 \| \| Soap \| Soft soap \| 2.6 kg \| 0.39 \| \| Linen \| - \| 5 m \| 1.35 \| \| Candles \| - \| 2.6 kg \| 1.86 \| \| Lamp oil \| Petroleum \| 2.6 L \| 0.31 \| \| Fuel \| Coal \| 5.0 M BTU \| 1.70 ^b^ \| \| Rent \|  \| 5% allowance \| 3.62 \| \| *Total* \|  \|  \| *81.60* \| |
| ^a^ The original amount of bread in the respectability basket is 234 kilograms. I raised it to 294 kilograms to come to a daily caloric total of 2,900, as set by the FAO.  ^b^ 100 kilogram of coal is 2.65 M BTU. I set the coal needs per basket at 200 kilograms.  Sources: Allen, *Amsterdam;* Allen, *The great divergence*, p. 421; van Riel, *Prices*. |

*Regional price differences*

To account for regional variation in prices, I have used Arthur van Riel’s recent dissertation. Van Riel calculated the costs of living as a percentage of the national average for each province as well as for rural and urban municipalities (Table S5.1). I calculated the rural costs of living (on a national level) and the urban costs of living in the province of Overijssel (where the region of Twente is situated). Note that van Riel did extensive research on household consumption in the Netherlands and that his results would be a more realistic deflator of household income. However, I choose to use Allen’s consumption baskets to make my results comparable with previous research on Dutch living standards.

| Table S5.2. Regional costs of living (in percentages of the national average) |
| --- |
| \|  \| urban costs of living \| rural costs of living \| provincial average \| urban-rural gap \| urban-provincial gap \| \| --- \| --- \| --- \| --- \| --- \| --- \| \| Groningen \| 115 \| 95.6 \| 96.5 \| 20.4 \| 19.2 \| \| Friesland \| 114.5 \| 98.7 \| 99.4 \| 16 \| 15.2 \| \| Drenthe \| 91.8 \| 96.8 \| 98.2 \| -5.2 \| -6.6 \| \| Overijssel \| 105.7 \| 95.9 \| 97.1 \| 10.2 \| 8.8 \| \| Gelderland \| 112.7 \| 99 \| 99.9 \| 13.9 \| 12.9 \| \| Utrecht \| 113.6 \| 100.5 \| 101.5 \| 13.1 \| 11.9 \| \| Noord-Holland \| 119.1 \| 107.2 \| 108.1 \| 11.1 \| 10.1 \| \| Zuid-Holland \| 117.5 \| 102.1 \| 103.5 \| 15.1 \| 13.5 \| \| Zeeland \| 113.2 \| 98 \| 99.6 \| 15.5 \| 13.6 \| \| Noord-Brabant \| 107.7 \| 94.6 \| 95.4 \| 13.8 \| 12.9 \| \| Limburg \| 103 \| 100.4 \| 100.7 \| 2.6 \| 2.3 \| \| **Average values** \| **110.3** \| **99** \| **100** \| **11.5** \| **10.4** \| \| Municipalities \| 34 \| 314 \| 348 \|  \|  \| |
| The table is taken from van Riel, *Trials of convergence*. Original title: *Urban-rural cost of living differentials by province based on the 1913 post- and telegraph services inquiry (348 municipal observations; overall provincial average = 100)*. |

| Table S5.3. Price of annual subsistence and respectability basket in guilders, 1910 |
| --- |
| \| **Basket** \| **Amsterdam** \| **National average** \| **Rural** \| **Urban (Overijssel)** \| \| --- \| --- \| --- \| --- \| --- \| \| Subsistence \| 36.50 \| 30.65 \| 30.34 \| 32.40 \| \| Respectability \| - \| 81.60 \| 80.78 \| 86.25 \| |
| Sources: Allen, *Amsterdam;* van Riel, *Trials of convergence*. |

*Consumption units*

To determine the consumptive needs of households at any stage of the life-cycle, we need to know how many consumption units or ‘male equivalents’ were present at any given time. As historians before me have done, I follow the age and gender-specific caloric requirements of the FAO. The baseline consuming unit caloric intake was set at 2,900 kcal per day. Note that pregnant and lactating women require more calories, which is included in the analysis.

| Table S5.4. Age- and gender-specific energy requirements |
| --- |
| \|  \| **Men** \| \| **Women** \| \| \| --- \| --- \| --- \| --- \| --- \| \| *Age* \| *Daily energy requirement (kcal/day)* \| *Consuming unit equivalent* \| *Daily energy requirement (kcal/day)* \| *Consuming unit equivalent* \| \| 0-1^a^ \| 948 \| 0 \| 865 \| 0 \| \| 1–2 \| 948 \| 0.33 \| 865 \| 0.3 \| \| 2–3 \| 1,129 \| 0.39 \| 1,047 \| 0.36 \| \| 3–4 \| 1,252 \| 0.43 \| 1,156 \| 0.40 \| \| 4–5 \| 1,360 \| 0.47 \| 1,241 \| 0.43 \| \| 5–6 \| 1,467 \| 0.51 \| 1,330 \| 0.46 \| \| 6–7 \| 1,573 \| 0.54 \| 1,428 \| 0.49 \| \| 7–8 \| 1,692 \| 0.58 \| 1,554 \| 0.54 \| \| 8–9 \| 1,830 \| 0.63 \| 1,698 \| 0.59 \| \| 9–10 \| 1,978 \| 0.68 \| 1,854 \| 0.64 \| \| 10–11 \| 2,150 \| 0.74 \| 2,006 \| 0.69 \| \| 11–12 \| 2,341 \| 0.81 \| 2,149 \| 0.74 \| \| 12–13 \| 2,548 \| 0.88 \| 2,276 \| 0.78 \| \| 13–14 \| 2,770 \| 0.96 \| 2,379 \| 0.82 \| \| 14–15 \| 2,990 \| 1.03 \| 2,449 \| 0.84 \| \| 15–16 \| 3,178 \| 1.10 \| 2,491 \| 0.86 \| \| 16–17 \| 3,322 \| 1.15 \| 2,503 \| 0.86 \| \| 17–18 \| 3,410 \| 1.18 \| 2,503 \| 0.86 \| \| >18 \| 3,410 \| 1.18 \| 2,503 \| 0.86 \| \| Pregnant^b^ \|  \|  \| 2,785 \| 0.96 \| \| Lactating \|  \|  \| 3,178 \| 1.10 \| |
| ^a^ The consuming unit equivalent is set at 0 for new-borns, assuming they were breastfed during their first year of life. This might result in a slight underestimation of the costs since they did require non-food items.  ^b^ The additional amount of calories for pregnant women is the average of the three trimesters. |

**Appendix S6.** **Agricultural household budgets**

The quantitative information in the source only provided information on the number of people younger and older than 15. In the qualitative information about each household the number of children and the range of their age was mentioned. For instance, for household number 22 it is reported that there were four children younger than 10. The gender of these children is never mentioned. Determining the number of consumption units in each household thus required making some assumptions and creativity. In order not to overstate the welfare ratios of these households, I assumed that all children were boys. Furthermore, if an age range was mentioned, as opposed to the exact age of all children, I interpolated the information. Thus, four children from 12-22 years old were assigned the ages of 12, 15, 18, and 22.

Note that the qualitative and quantitative information about the total number of people did not match for households 15 and 18. I used the qualitative information for determining the number of consumption units.

| Table S6.1. Household composition, income (in guilders per year), and welfare ratios of 25 agricultural households, 1913 |
| --- |
| \|  \| **Household members** \| \|  \| **Income (in guilders)** \| \| **Welfare ratios (subsistence)** \| \| **Welfare ratios (respectability)** \| \| \| --- \| --- \| --- \| --- \| --- \| --- \| --- \| --- \| --- \| --- \| \| *Nr.* \| *People >15* \| *People <15* \| *Total baskets needed* \| *Wage husband* \| *Total income* \| *Wage husband* \| *Total income* \| *Wage husband* \| *Total income* \| \| 1 \| 2 \| 4 \| 3.89 \| 455.63 \| 470.06 \| 0.40 \| 4.69 \| 0.16 \| 1.85 \| \| 2 \| 2 \| 5 \| 5.30 \| 495.11 \| 538.66 \| 0.42 \| 4.30 \| 0.17 \| 1.70 \| \| 3 \| 2 \| 4 \| 4.07 \| 442.24 \| 507.61 \| 0.96 \| 3.78 \| 0.38 \| 1.49 \| \| 4 \| 2 \| 2 \| 3.14 \| 215.30 \| 397.31 \| 1.01 \| 4.82 \| 0.40 \| 1.90 \| \| 5 \| 5 \| 1 \| 6.19 \| 82.59 \| 847.92 \| 1.24 \| 3.20 \| 0.49 \| 1.26 \| \| 6 \| 4 \| 2 \| 6.38 \| 480.61 \| 1772.16 \| 1.30 \| 4.66 \| 0.51 \| 1.84 \| \| 7 \| 2 \| 5 \| 5.05 \| 162.84 \| 775.56 \| 1.63 \| 8.84 \| 0.64 \| 3.49 \| \| 8 \| 2 \| 5 \| 5.54 \| 481.57 \| 733.65 \| 1.70 \| 5.35 \| 0.67 \| 2.11 \| \| 9 \| 3 \| 3 \| 4.90 \| 445.25 \| 767.24 \| 1.74 \| 4.82 \| 0.69 \| 1.90 \| \| 10 \| 2 \| 6 \| 5.31 \| 473.36 \| 607.45 \| 1.99 \| 5.66 \| 0.78 \| 2.23 \| \| 11 \| 3 \| 5 \| 6.57 \| 363.70 \| 1008.77 \| 2.15 \| 3.97 \| 0.85 \| 1.57 \| \| 12 \| 2 \| 2 \| 2.80 \| 368.54 \| 728.93 \| 2.28 \| 6.41 \| 0.90 \| 2.53 \| \| 13 \| 2 \| 1 \| 2.62 \| 136.36 \| 737.91 \| 2.36 \| 8.72 \| 0.93 \| 3.44 \| \| 14 \| 7 \| 5 \| 11.02 \| 596.74 \| 1878.64 \| 2.73 \| 4.16 \| 1.08 \| 1.64 \| \| 15 \| 2 \| 1 \| 2.76 \| 464.50 \| 571.45 \| 2.80 \| 3.59 \| 1.10 \| 1.42 \| \| 16 \| 2 \| 6 \| 5.98 \| 182.60 \| 720.91 \| 2.85 \| 4.92 \| 1.12 \| 1.94 \| \| 17 \| 3 \| 3 \| 5.01 \| 207.70 \| 744.05 \| 2.93 \| 3.19 \| 1.16 \| 1.26 \| \| 18 \| 3 \| 3 \| 3.31 \| 240.87 \| 676.32 \| 3.41 \| 3.91 \| 1.35 \| 1.54 \| \| 20 \| 3 \| 6 \| 7.42 \| 293.00 \| 755.37 \| 3.43 \| 5.01 \| 1.35 \| 1.97 \| \| 21 \| 4 \| 1 \| 4.71 \| 298.60 \| 849.89 \| 3.51 \| 4.25 \| 1.39 \| 1.68 \| \| 22 \| 2 \| 4 \| 4.30 \| 470.00 \| 685.64 \| 3.55 \| 4.49 \| 1.40 \| 1.77 \| \| 23 \| 2 \| 3 \| 3.75 \| 419.75 \| 507.97 \| 3.68 \| 3.79 \| 1.45 \| 1.50 \| \| 24 \| 2 \| 5 \| 4.32 \| 55.41 \| 646.00 \| 4.13 \| 8.17 \| 1.63 \| 3.22 \| \| 25 \| 3 \| 3 \| 4.17 \| 471.40 \| 596.23 \| 5.26 \| 5.89 \| 2.07 \| 2.32 \| \| 27 \| 2 \| 3 \| 3.31 \| 554.80 \| 620.61 \| 5.28 \| 6.50 \| 2.08 \| 2.56 \| \| ***Av.*** \| ***2.72*** \| ***3.52*** \| ***4.87*** \| ***354.34*** \| ***765.85*** \|  \|  \|  \|  \| |
| Sources: Allen, *Amsterdam;* Koninklijke Nederlandsche Landbouwvereeniging, 'Bijzondere catalogus', Appendix IV; van Riel, *Trials of convergence*. |

| Figure S6.1. Welfare ratios of 25 agricultural households at the respectability level, ca. 1910 |
| --- |
|  |
| Source: Koninklijke Nederlandsche Landbouwvereeniging, *Bijzondere catalogus*. |

**Appendix S7.** **Historical Sample of the Netherlands: average number of children and child mortality**

To examine the average number of children per household, I have selected all the households in the HSN database of women who were born in the 1870s (N = 648). The results are summarized in the table below and classified into four categories, based on the occupation of the husband. The median number of children was 4 and the average almost 5 in the households of each category, except for the group with a husbands with an unknown occupation.

| Table S7.1. Number of children and cases of infant mortality in 648 Dutch households per sector |
| --- |
| \| **Sector** \| **Number of mothers** \| **Number of children per mother (mean)** \| **Number of children per mother (median)** \| **Total children** \| **Child mortality ^a^** \| \| --- \| --- \| --- \| --- \| --- \| --- \| \| Agriculture \| 70 \| 4.8 \| 4 \| 334 \| 21 \| \| Industry \| 248 \| 4.9 \| 4 \| 1,216 \| 85 \| \| Services \| 164 \| 4.6 \| 4 \| 749 \| 58 \| \| Unknown \| 166 \| 5.7 \| 5 \| 954 \| 72 \| \| *Total* \| *648* \| *5.0* \| *4* \| *3,253* \| *236* \| |
| Source: HSN  ^a^ Number of children who died in the same year or the year after they were born. |

**Appendix S8. Income and consumption during the life-cycle**

| Table S8.1. Life-cycle welfare ratios of agricultural households (subsistence level) |
| --- |
| \| A.  Life-cycle year \| B.  Number of consumption units \| C.  Annual costs baskets  (B * *f*31.86) \| D.  Income \| E.  Welfare ratio  (D/C) \| \| --- \| --- \| --- \| --- \| --- \| \| 1 \| 2.14 \| 68.19 \| 838.19 \| 12.29 \| \| 2 \| 2.28 \| 72.51 \| 819.19 \| 11.30 \| \| 3 \| 2.44 \| 77.75 \| 838.19 \| 10.78 \| \| 4 \| 2.64 \| 83.98 \| 819.19 \| 9.75 \| \| 5 \| 2.74 \| 87.30 \| 857.19 \| 9.82 \| \| 6 \| 2.83 \| 90.16 \| 857.19 \| 9.51 \| \| 7 \| 2.90 \| 92.39 \| 857.19 \| 9.28 \| \| 8 \| 2.96 \| 94.31 \| 857.19 \| 9.09 \| \| 9 \| 3.14 \| 100.05 \| 838.19 \| 8.38 \| \| 10 \| 3.36 \| 106.92 \| 819.19 \| 7.66 \| \| 11 \| 3.52 \| 112.15 \| 857.19 \| 7.64 \| \| 12 \| 3.78 \| 120.44 \| 838.19 \| 6.96 \| \| 13 \| 4.06 \| 129.22 \| 819.19 \| 6.34 \| \| 14 \| 4.27 \| 136.04 \| 894.19 \| 6.57 \| \| 15 \| 4.45 \| 141.78 \| 894.19 \| 6.31 \| \| 16 \| 4.58 \| 145.92 \| 931.19 \| 6.38 \| \| 17 \| 4.83 \| 153.89 \| 912.19 \| 5.93 \| \| 18 \| 5.08 \| 161.72 \| 893.19 \| 5.52 \| \| 19 \| 5.27 \| 167.90 \| 931.19 \| 5.55 \| \| 20 \| 4.56 \| 145.28 \| 894.19 \| 6.15 \| \| 21 \| 4.70 \| 149.74 \| 894.19 \| 5.97 \| \| 22 \| 3.97 \| 126.48 \| 894.19 \| 7.07 \| \| 23 \| 4.11 \| 130.94 \| 894.19 \| 6.83 \| \| 24 \| 4.23 \| 134.77 \| 894.19 \| 6.64 \| \| 25 \| 4.36 \| 138.91 \| 931.19 \| 6.70 \| \| 26 \| 4.49 \| 143.05 \| 931.19 \| 6.51 \| \| 27 \| 4.61 \| 146.87 \| 931.19 \| 6.34 \| \| 28 \| 3.88 \| 123.62 \| 894.19 \| 7.23 \| \| 29 \| 4.00 \| 127.44 \| 894.19 \| 7.02 \| \| 30 \| 4.10 \| 130.63 \| 931.19 \| 7.13 \| \| 31 \| 3.00 \| 95.58 \| 894.19 \| 9.36 \| \| 32 \| 3.07 \| 97.81 \| 894.19 \| 9.14 \| \| 33 \| 3.14 \| 100.04 \| 894.19 \| 8.94 \| \| 34 \| 3.19 \| 101.63 \| 894.19 \| 8.80 \| \| 35 \| 3.22 \| 102.59 \| 894.19 \| 8.72 \| \| 36 \| 2.04 \| 64.99 \| 857.19 \| 13.19 \| |
|  |

| Table S8.2. Life-cycle welfare ratios of textile households (subsistence level) |
| --- |
| \| A.  Life-cycle year \| B.  Number of consumption units \| C.  Annual costs baskets  (B * *f*34.02) \| D.  Income \| E.  Welfare ratio  (D/C) \| \| --- \| --- \| --- \| --- \| --- \| \| 1 \| 2.14 \| 72.81 \| 773.93 \| 10.6 \| \| 2 \| 2.14 \| 72.81 \| 773.93 \| 10.6 \| \| 3 \| 2.28 \| 77.42 \| 689.10 \| 8.9 \| \| 4 \| 2.37 \| 80.63 \| 689.10 \| 8.5 \| \| 5 \| 2.43 \| 82.67 \| 689.10 \| 8.3 \| \| 6 \| 2.57 \| 87.44 \| 689.10 \| 7.9 \| \| 7 \| 2.75 \| 93.41 \| 689.10 \| 7.4 \| \| 8 \| 2.88 \| 97.98 \| 692.24 \| 7.1 \| \| 9 \| 3.07 \| 104.45 \| 692.24 \| 6.6 \| \| 10 \| 3.29 \| 111.79 \| 692.24 \| 6.2 \| \| 11 \| 3.14 \| 106.82 \| 692.24 \| 6.5 \| \| 12 \| 3.33 \| 113.30 \| 692.24 \| 6.1 \| \| 13 \| 3.56 \| 120.97 \| 702.38 \| 5.8 \| \| 14 \| 3.73 \| 126.89 \| 702.38 \| 5.5 \| \| 15 \| 3.91 \| 133.02 \| 808.46 \| 6.1 \| \| 16 \| 4.08 \| 138.80 \| 840.70 \| 6.1 \| \| 17 \| 4.34 \| 147.66 \| 908.30 \| 6.2 \| \| 18 \| 4.65 \| 158.05 \| 1011.98 \| 6.4 \| \| 19 \| 4.86 \| 165.34 \| 1118.06 \| 6.8 \| \| 20 \| 5.08 \| 172.82 \| 1150.30 \| 6.7 \| \| 21 \| 4.06 \| 138.12 \| 1011.98 \| 7.3 \| \| 22 \| 4.21 \| 143.22 \| 1011.98 \| 7.1 \| \| 23 \| 4.34 \| 147.65 \| 868.63 \| 5.9 \| \| 24 \| 4.45 \| 151.39 \| 868.63 \| 5.7 \| \| 25 \| 3.36 \| 114.31 \| 768.79 \| 6.7 \| \| 26 \| 3.45 \| 117.37 \| 801.03 \| 6.8 \| \| 27 \| 3.52 \| 119.75 \| 868.63 \| 7.3 \| \| 28 \| 3.59 \| 122.13 \| 817.67 \| 6.7 \| \| 29 \| 3.64 \| 123.83 \| 817.67 \| 6.6 \| \| 30 \| 3.68 \| 125.19 \| 923.75 \| 7.4 \| \| 31 \| 2.86 \| 97.30 \| 750.07 \| 7.7 \| \| 32 \| 2.88 \| 97.98 \| 817.67 \| 8.3 \| \| 33 \| 2.90 \| 98.66 \| 843.67 \| 8.6 \| \| 34 \| 2.90 \| 98.66 \| 843.67 \| 8.6 \| \| 35 \| 2.90 \| 98.66 \| 843.67 \| 8.6 \| \| 36 \| 2.04 \| 69.40 \| 637.75 \| 9.2 \| |
|  |

| Table S8.3. Life-cycle welfare ratios of agricultural households (respectability level) |
| --- |
| \| A.  Life-cycle year \| B.  Number of consumption units \| C.  Annual costs baskets  (B * *f*80.78) \| D.  Income \| E.  Welfare ratio  (D/C) \| \| --- \| --- \| --- \| --- \| --- \| \| 1 \| 2.14 \| 172.87 \| 838.19 \| 4.85 \| \| 2 \| 2.28 \| 184.18 \| 819.19 \| 4.46 \| \| 3 \| 2.44 \| 197.10 \| 838.19 \| 4.25 \| \| 4 \| 2.64 \| 213.26 \| 819.19 \| 3.85 \| \| 5 \| 2.74 \| 221.34 \| 857.19 \| 3.87 \| \| 6 \| 2.83 \| 228.61 \| 857.19 \| 3.75 \| \| 7 \| 2.90 \| 234.26 \| 857.19 \| 3.66 \| \| 8 \| 2.96 \| 239.11 \| 857.19 \| 3.58 \| \| 9 \| 3.14 \| 253.65 \| 838.19 \| 3.30 \| \| 10 \| 3.36 \| 271.42 \| 819.19 \| 3.02 \| \| 11 \| 3.52 \| 284.35 \| 857.19 \| 3.01 \| \| 12 \| 3.78 \| 305.35 \| 838.19 \| 2.74 \| \| 13 \| 4.06 \| 327.97 \| 819.19 \| 2.50 \| \| 14 \| 4.27 \| 344.93 \| 894.19 \| 2.59 \| \| 15 \| 4.45 \| 359.47 \| 894.19 \| 2.49 \| \| 16 \| 4.58 \| 369.97 \| 931.19 \| 2.52 \| \| 17 \| 4.83 \| 390.17 \| 912.19 \| 2.34 \| \| 18 \| 5.08 \| 410.36 \| 893.19 \| 2.18 \| \| 19 \| 5.27 \| 425.71 \| 931.19 \| 2.19 \| \| 20 \| 4.56 \| 368.36 \| 894.19 \| 2.43 \| \| 21 \| 4.70 \| 379.67 \| 894.19 \| 2.36 \| \| 22 \| 3.97 \| 320.70 \| 894.19 \| 2.79 \| \| 23 \| 4.11 \| 332.01 \| 894.19 \| 2.69 \| \| 24 \| 4.23 \| 341.70 \| 894.19 \| 2.62 \| \| 25 \| 4.36 \| 352.20 \| 931.19 \| 2.64 \| \| 26 \| 4.49 \| 362.70 \| 931.19 \| 2.57 \| \| 27 \| 4.61 \| 372.40 \| 931.19 \| 2.50 \| \| 28 \| 3.88 \| 313.43 \| 894.19 \| 2.85 \| \| 29 \| 4.00 \| 323.12 \| 894.19 \| 2.77 \| \| 30 \| 4.10 \| 331.20 \| 931.19 \| 2.81 \| \| 31 \| 3.00 \| 242.34 \| 894.19 \| 3.69 \| \| 32 \| 3.07 \| 247.99 \| 894.19 \| 3.61 \| \| 33 \| 3.14 \| 253.65 \| 894.19 \| 3.53 \| \| 34 \| 3.19 \| 257.69 \| 894.19 \| 3.47 \| \| 35 \| 3.22 \| 260.11 \| 894.19 \| 3.44 \| \| 36 \| 2.04 \| 164.79 \| 857.19 \| 5.20 \| |
|  |

| Table S8.4. Life-cycle welfare ratios of textile households (respectability level) |
| --- |
| \| A.  Life-cycle year \| B.  Number of consumption units \| C.  Annual costs baskets  (B * *f*86.25) \| D.  Income \| E.  Welfare ratio  (D/C) \| \| --- \| --- \| --- \| --- \| --- \| \| 1 \| 2.14 \| 184.60 \| 773.93 \| 4.2 \| \| 2 \| 2.14 \| 184.60 \| 773.93 \| 4.2 \| \| 3 \| 2.28 \| 196.29 \| 689.10 \| 3.5 \| \| 4 \| 2.37 \| 204.41 \| 689.10 \| 3.4 \| \| 5 \| 2.43 \| 209.59 \| 689.10 \| 3.3 \| \| 6 \| 2.57 \| 221.69 \| 689.10 \| 3.1 \| \| 7 \| 2.75 \| 236.83 \| 689.10 \| 2.9 \| \| 8 \| 2.88 \| 248.40 \| 692.24 \| 2.8 \| \| 9 \| 3.07 \| 264.82 \| 692.24 \| 2.6 \| \| 10 \| 3.29 \| 283.41 \| 692.24 \| 2.4 \| \| 11 \| 3.14 \| 270.83 \| 692.24 \| 2.6 \| \| 12 \| 3.33 \| 287.24 \| 692.24 \| 2.4 \| \| 13 \| 3.56 \| 306.69 \| 702.38 \| 2.3 \| \| 14 \| 3.73 \| 321.71 \| 702.38 \| 2.2 \| \| 15 \| 3.91 \| 337.24 \| 808.46 \| 2.4 \| \| 16 \| 4.08 \| 351.90 \| 840.70 \| 2.4 \| \| 17 \| 4.34 \| 374.35 \| 908.30 \| 2.4 \| \| 18 \| 4.65 \| 400.71 \| 1011.98 \| 2.5 \| \| 19 \| 4.86 \| 419.18 \| 1118.06 \| 2.7 \| \| 20 \| 5.08 \| 438.15 \| 1150.30 \| 2.6 \| \| 21 \| 4.06 \| 350.18 \| 1011.98 \| 2.9 \| \| 22 \| 4.21 \| 363.11 \| 1011.98 \| 2.8 \| \| 23 \| 4.34 \| 374.33 \| 868.63 \| 2.3 \| \| 24 \| 4.45 \| 383.81 \| 868.63 \| 2.3 \| \| 25 \| 3.36 \| 289.80 \| 768.79 \| 2.7 \| \| 26 \| 3.45 \| 297.56 \| 801.03 \| 2.7 \| \| 27 \| 3.52 \| 303.60 \| 868.63 \| 2.9 \| \| 28 \| 3.59 \| 309.64 \| 817.67 \| 2.6 \| \| 29 \| 3.64 \| 313.95 \| 817.67 \| 2.6 \| \| 30 \| 3.68 \| 317.40 \| 923.75 \| 2.9 \| \| 31 \| 2.86 \| 246.68 \| 750.07 \| 3.0 \| \| 32 \| 2.88 \| 248.40 \| 817.67 \| 3.3 \| \| 33 \| 2.90 \| 250.13 \| 843.67 \| 3.4 \| \| 34 \| 2.90 \| 250.13 \| 843.67 \| 3.4 \| \| 35 \| 2.90 \| 250.13 \| 843.67 \| 3.4 \| \| 36 \| 2.04 \| 175.95 \| 637.75 \| 3.6 \| |
|  |

References

Allen, R. C., ‘Amsterdam’ https://www.nuffield.ox.ac.uk/people/sites/allen-research-pages/ (accessed on 24 July, 2019).

Allen, R. C., ‘The Great Divergence in European wages and prices from the Middle Ages to the First World War’, *Explorations in Economic History* 38 (2001), pp. 411–47.

Bieleman, J., *Boeren op het Drentse zand 1600-1910. Een nieuwe visie op de oude landbouw* (Wageningen, 1987).

Centraal Bureau voor de Statistiek, 'Statistiek van de loonen der volgens de ongevallenwet 1901 verzekerde werklieden in de textielindustrie in 1908', *Bijdragen tot de Statistiek van Nederland* (1908).

Departement van Landbouw Nijverheid en Handel, *Verslag over den landbouw in Nederland over 1911* (The Hague, 1912).

Directie van den Arbeid, *Onderzoekingen naar de toestanden in de Nederlandsche huisindustrie*, deel I: *Voedings- en genotmiddelen* (The Hague, 1914).

Directie van den Arbeid, *Onderzoekingen naar de toestanden in de Nederlandsche huisindustrie*, deel II: *Diamantbewerking, drukkersbedrijf, chemische industrie, hout- en stroobewerking, metaalbewerking, papierbewerking, schoenmakers* (The Hague, 1914).

Directie van den Arbeid, *Onderzoekingen naar de toestanden in de Nederlandsche huisindustrie*, deel III: *Textielindustrie – kleeding en reiniging* (The Hague, 1914).

Koninklijke Nederlandsche Landbouwvereeniging, *Nationale en internationale landbouwtentoonstelling te ’s-gravenhage in september 1913. Bijzondere catalogus* (The Hague, 1913).

Posthumus, N. W., *Huisindustrie in Nederland. Loon- en arbeidsverhoudingen. Catalogus der voorwerpen aanwezig op de Nederlandsche tentoonstelling van huisindustrie te Amsterdam* (Amsterdam, 1909).

Reudink, G., ‘Nijverheidsstatistiek Struve en Bekaar’ (1994) http://www.neha.nl/struve/ (accessed on May, 2019).

van Riel, A., ‘Prices of consumer and producer goods, 1800–1913’ http://www.iisg.nl/hpw/brannex.php (accessed on January, 2020).

van Riel, A., ‘Trials of convergence: Prices, markets and industrialization in the Netherlands, 1800-1913’ (unpub. Ph.D. thesis, Utrecht University, 2018).

1. Cited in: Bieleman, Boeren op het Drentse zand, p. 512. [↑](#footnote-ref-1)
2. Bieleman, Boeren op het Drentse zand, p. 510. [↑](#footnote-ref-2)
3. Independent farmers owned 20-25 chickens. [↑](#footnote-ref-3)
4. Bieleman, Boeren op het Drentse zand, pp. 514-6. [↑](#footnote-ref-4)
5. Directie van den Arbeid, Onderzoekingen (part I); Directie van den Arbeid, Onderzoekingen (part II); Directie van den Arbeid, Onderzoekingen (part III). [↑](#footnote-ref-5)
6. The database can be found on this website: <http://www.iisg.nl/hpw/brannex.php> [↑](#footnote-ref-6)
